# Waldenstrom macroglobulinemia cells devoid of *BTK^C481S^* or *CXCR4^WHIM-like^* mutations acquire resistance to ibrutinib through upregulation of Bcl-2 and AKT resulting in vulnerability towards venetoclax or MK2206 treatment

**DOI:** 10.1038/bcj.2017.40

**Published:** 2017-05-26

**Authors:** A Paulus, S Akhtar, H Yousaf, A Manna, S M Paulus, Y Bashir, T R Caulfield, M Kuranz-Blake, K Chitta, X Wang, Y Asmann, R Hudec, W Springer, S Ailawadhi, A Chanan-Khan

**Affiliations:** 1 Department of Cancer Biology, Mayo Clinic, Jacksonville, FL, USA; 2 Division of Hematology and Oncology, Mayo Clinic, Jacksonville, FL USA; 3 Department of Molecular Neuroscience, Mayo Clinic, Jacksonville, FL, USA; 4 Department of Health Sciences Research, Mayo Clinic, Jacksonville, FL USA

## Abstract

Although ibrutinib is highly effective in Waldenstrom macroglobulinemia (WM), no complete remissions in WM patients treated with ibrutinib have been reported to date. Moreover, ibrutinib-resistant disease is being steadily reported and is associated with dismal clinical outcome (overall survival of 2.9–3.1 months). To understand mechanisms of ibrutinib resistance in WM, we established ibrutinib-resistant *in vitro* models using validated WM cell lines. Characterization of these models revealed the absence of BTK^C481S^ and CXCR4^WHIM-like^ mutations. BTK-mediated signaling was found to be highly attenuated accompanied by a shift in PI3K/AKT and apoptosis regulation-associated genes/proteins. Cytotoxicity studies using the AKT inhibitor, MK2206±ibrutinib, and the Bcl-2-specific inhibitor, venetoclax±ibrutinib, demonstrated synergistic loss of cell viability when either MK22016 or venetoclax were used in combination with ibrutinib. Our findings demonstrate that induction of ibrutinib resistance in WM cells can arise independent of BTK^C481S^ and CXCR4^WHIM-like^ mutations and sustained pressure from ibrutinib appears to activate compensatory AKT signaling as well as reshuffling of Bcl-2 family proteins for maintenance of cell survival. Combination treatment demonstrated greater (and synergistic) antitumor effect and provides rationale for development of therapeutic strategies encompassing venetoclax+ibrutinib or PI3K/AKT inhibitors+ibrutinib in ibrutinib-resistant WM.

## Introduction

Waldenstrom macroglobulinemia (WM), a rare non-Hodgkin lymphoma variant, is characterized by unrestrained clonal proliferation of lymphoplasmacytic cells in the bone marrow and lymphoid tissue (lymph nodes, spleen). Patients usually present with cytopenias, lymphadenopathy and/or hepatosplenomegaly.^1^ In addition, WM cells produce and secrete excessive amounts of monoclonal immunoglobulin M (IgM), which can cause hyperviscosity syndrome and its associated complications. Therapeutic strategies have been extrapolated from other low-grade non-Hodgkin lymphoma and until very recently no drug had specifically secured approval in WM.^2^

Ibrutinib, a first-in-class Brutons tyrosine kinase (BTK) inhibitor, is the first drug to gain Food and Drug Administration approval for treatment of WM and represents a milestone for patients suffering from this malignancy. In a phase II trial, relapsed or refractory WM patients who received ibrutinib demonstrated an overall response rate of 90.5%, with a major response rate of 70.5%. Estimated progression-free and overall survival (OS) at 24 months of treatment was 69.1% (95% confidence interval (CI): 53.2–80.5) and 95.2% (95% CI: 86.0–98.4), respectively.^3^ However, no complete remissions were observed, indicating the WM cell’s ability to maintain their survival under ibrutinib-induced stress.

Despite the clinical benefit derived by patients treated with ibrutinib, undoubtedly the phenomenon of resistance to its effects is increasingly being reported in chronic lymphocytic leukemia (CLL), mantle cell lymphoma (MCL) and also WM (malignancies for which ibrutinib is currently approved).^4, 5, 6, 7, 8, 9, 10, 11^ Biologically this reflects the malignant tumor clone’s ability to survive sustained BTK inhibition and indicates the lack of curative potential at least with ibrutinib monotherapy. Indeed, ibrutinib-resistant disease is now consistently reported with fatal outcome, with median OS of CLL and MCL patients who relapse on ibrutinib being ~3.1 and 2.9 months, respectively.^12, 13^ Although OS data for postibrutinib relapse WM patients is not yet available, it is anticipated that when these patients relapse (or become refractory to ibrutinib), their survival outcome may follow a similar dismal clinical course. Our laboratory efforts preemptively have tried to address this problem through development of unique models to interrogate the biology of ibrutinib resistance in WM in a quest to become prepared for potential salvage approaches.^14, 15, 16^

Mechanistically, ibrutinib binds the Cys^481^ residue of the BTK kinase domain-active site and blocks autophosphorylation required for BTK activation.^17^ In CLL and MCL patients, it has been reported that a cysteine-to-serine point mutation at residue 481 (C481S) in the allosteric inhibitory segment of *BTK* diminishes ibrutinib’s antitumor activity.^6, 8, 18^ Similar observation has not yet been confirmed in WM patients, and even in CLL and MCL, *BTK^C481S^* is not universally noted in all patients who develop ibrutinib resistance.^19, 20^ In WM, *CXCR4^WHIM-like^* mutations have been suggested as determinants of response to ibrutinib. However, the observation that 38% of WM patients who are *MYD88_L265P+_/CXCR4^WHIM+^* exhibit suboptimal response (i.e. less than major response) vs 62% of *MYD88_L265P+_/CXCR4^WHIM+^* patients who demonstrate major responses suggests that mechanisms other than *CXCR4^WHIM-like^* mutation must account for ibrutinib resistance.^11^ Considering ibrutinib is the only approved therapeutic for WM, interrogation of the molecular mechanisms of resistance to ibrutinib in WM is of paramount importance to unveil new therapeutic opportunities in patients who have relapsed or become refractory to ibrutinib therapy.^21^

## Materials and methods

### Cell lines, cell culture and reagents

WM cell lines and their corresponding ibrutinib-resistant clones, developed in our laboratory, were used in experiments. All cell lines were cultured in RPMI-1640 containing 10% fetal bovine serum, penicillin (100 U/ml) and streptomycin (100 μg/ml). Cell viability was always maintained at >90% and was measured by trypan blue exclusion assay using ViCell-XR viability counter (Beckman-Coulter, Indianapolis, IN, USA). RPMI, penicillin, streptomycin, tetramethylrhodamine, methyl ester (TMRM) and fetal bovine serum were purchased from Life technologies (Carlsbad, CA, USA). Ibrutinib, MK2206 and ABT-199 (venetoclax) were purchased from Sellekhem (Houston, TX, USA). Annexin-V/Propidium Iodide Apoptosis Staining Kit was purchased from BD Biosciences (San Jose, CA, USA).

### Cell proliferation, viability and apoptosis assays

MTS assay (Molecular Probes, ThermoFisher Scientific, Rockford, IL, USA) or the CellTiter Glo Luminescent Cell Assay (Promega, Madison, WI, USA) were used to establish proliferation and viability of tumor cells, under previously reported conditions.^15, 22^ Apoptosis was measured by annexin-V and propidium iodide staining of cells followed by flow cytometry analysis. The conditions and method for these assays have been previously described by us.^15^ Drug combination synergy, additivity or antagonism were determined using the CompuSyn Software (ComboSyn Inc., Paramus, NJ, USA), which uses the Chou-Talalay principle.^23^ Details for this analysis are presented in the Supplementary Material and methods.

### Determination of MOMP

Cells were treated with venetoclax for 12 h and assessed for mitochondrial outer membrane permeability (MOMP) using TMRM in a manner similar to that previously reported by us.^15, 22, 24, 25^

### Cell respirometry assay

Mitochondrial oxygen consumption rate was measured using the Seahorse XF96 Flux Analyzer (Seahorse Bioscience, Billerica, MA, USA). BCWM.1 and BCWM.1/IR cells were collected by centrifugation (room temperature (RT), 10 min, 200 *g*) washed two times with custom-made no phenol red containing low-buffered non-bicarbonated Seahorse medium (pH 7.35): powder Dulbecco’s modified Eagle’s media supplemented with 10 mM glucose, 10 mM pyruvate and 2 mM l-glutamine (all from Sigma-Aldrich, St Louis, MO, USA). Cell counts were adjusted and 2.5 × 10^5^ vital cells per well were seeded on Seahorse 96-well plate previously coated with poly-d-lysine—in pentaplicates per each condition. Pelleted cells (room temperature, 1 min, 600 *g*) were further caped in CO_2_-free incubator for 1 h prior tobefore the experiment. The Seahorse program included a 20 min probe equilibration step followed by a 3 min mix, and 3 min measurement, which was repeated three times before and after each injection. Drugs were injected sequentially in final concentration as follows: 2 μM oligomycin, 1.5 μM carbonyl cyanide-4-phenylhydrazone and combination of rotenone and antimycin A each 0.5 μM (all from Sigma-Aldrich). After completion of the assay, the obtained oxygen consumption rate values were normalized to total protein content. Cells in each well were lysed in RIPA buffer: 50 mM Tris-Cl (pH 8.0), 150 mM NaCl, 0.1% sodium dodecyl sulfate, 0.5% deoxycholate and 1% NP-40 (all from Sigma-Aldrich) and protein content was determined using the BCA Protein Assay (ThermoFisher Scientific) according to the manufacturer’s recommendation. Normalized data were analyzed using Seahorse Wave software and Excel macro file XF Mito Stress Test Report Generator (Agilent Technologies, Santa Clara, CA, USA). Displayed data show mean values and standard error of the mean.

### Immunoblot analysis

Western blot was performed for specific cellular protein estimations as reported previously.^25^ Total protein extracts were prepared using RIPA lysis buffer alongside 0.2% protease and a phosphatase inhibitor cocktail (Sigma-Aldrich). Supernatant was collected for western blot analyses after centrifugation, and the protein content was measured by BCA protein assay reagent. Twenty micrograms of total protein were then boiled in Laemmli sample buffer, loaded onto 10% sodium dodecyl sulfate-polyacrylamide gel electrophoresis gels and transferred onto a nitrocellulose membrane. Blots were developed using chemiluminescence (ThermoFisher Scientific).

### Sanger sequencing of *BTK* and *CXCR4*

RNA was isolated from all WM cells, including ibrutinib-resistant cloens, using the miRCURY RNA Isolation Kit (300110; Exiqon, Woburn, MA, USA) and quantified using NanoDrop 3000 (Thermofisher Scientific). Using specific primer sequence for the whole gene, RNA was converted to cDNA by using SuperScript III One Step RT-PCR (12574030; Thermofisher Scientific). Primers for BTK and CXCR4 are included in the Supplementary Materials and methods.

### Cell surface antigen analysis

For staining of cell surface markers, WM cells were washed two times with cold phosphate-buffered saline (PBS) and suspended in 300 μl of binding buffer (PBS solution with 2% fetal bovine serum). Cells were divided into three tubes: unstained, isotype control and those with antibody against CD19 (MACS Miltenyi Biotech, San Diego CA, USA), κ-light-chain and λ-light-chain (Invitrogen, Life Technologies, Carlsbad, CA, USA). Five microliters of the respective antibody was added and cells were incubated for 30 min at room temperature. Tumor cells were washed two times with cold PBS and suspended in 100 μl of 4% paraformaldehyde in PBS solution (ThermoFisher Scientific), followed by analysis using a BD Accuri C6 Flow Cytometer (Agilent Technologies, Santa Clara, CA, USA). FCS Express 4 (De Novo Softwares, Glendale, CA, USA) was used to analyze the data.

### Cell cycle analysis

Cells were serum starved for 4 h and treated with the drug for 12 h under normal culture conditions (10% fetal bovine serum). One million cells per sample were washed two times with cold PBS (SH30256; GE Healthcare Life Sciences, Logan, UT, USA). Cells were later resuspended in 0.5% glucose solution in PBS; later, they were fixed by 5 ml of 70% ice-cold alcohol and incubated at −20° F overnight. Cells were spun down at 2500 r.p.m. for 5 min to decant the alcohol and then washed two times with 0.1% Triton X-100 solution. Cells were resuspended in 1 ml of PBS containing RNAase (100 μg/ml) and propidium iodide stain (50 μg/ml) solution (BD Biosciences) and incubated in water bath (37 °C) for 30 min. With a pipette, cells were resuspended and read on an Accuri C6 Flow Cytometer (BD Biosciences) at slow speed. Samples were analyzed using FCS Express 4 (De Novo Softwares).

### Human IgM measurement

To quantify human IgM secreted in the sera of WM cells, we used the E-80M Human IgM ELISA Kit (Immunology Consultants Laboratory, Portland, OR, USA) according to the manufacturer's instructions.

### Digital mRNA expression profiling (nCounter NanoString platform)

Focused mRNA profiling and informatics analysis was carried out using the NanoString nCounter Assay (NanoString, Seattle, WA, USA) to determine the expression of select cancer genes in WM cells treated with dimethyl sulfoxide (DMSO) or ibrutinib, as described previously.^22, 25, 26^

### Whole-exome sequencing

DNA samples from BCWM.1, MWCL-1 and RPCI-WM1 and their respective ibrutinib-resistant derivatives (BCWM.1/IR, MWCL-1/IR, RPCI-WM1/IR) were captured using SureSelect Human All Exon V5+UTRs from Agilent Technologies. Additional details are described in the Supplementary Materials and methods.

## Results

### Induction of ibrutinib resistance, independent of *BTK^C481S^* or *CXCR4^WHIM-like^* mutations

**Figure 1:**
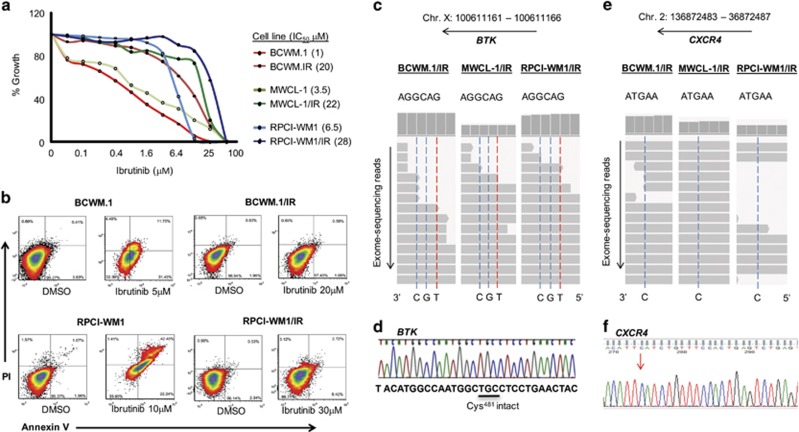
Establishment and characterization of ibrutinib-resistant cell lines demonstrates growth and apoptosis resistance without acquisition of *BTK^C481S^* or *CXCR4^WHIM-like^* mutations. Ibrutinib-resistant clones of established WM cell lines (*n*=3) were developed through chronic ibrutinib exposure over a period of 6 months. (**a**) A 72 h MTS assay was conducted to determine resistance of ibrutinib-resistant (/IR) WM cells vs WT parental cells. Median IC_50_ of ibrutinib-resistant cells was 22 μm (range, 20–28 μm), whereas median IC_50_ of WT WM cells was 3.5 μm (range, 1–6.5 μm). (**b**) All WM cell lines (WT and ibrutinib-resistant, *n*=6) were treated with DMSO or ibrutinib at 5, 10, 20 and 30 μm concentrations for 24 h and stained with annexin-V and propidium iodide, followed by flow cytometry to examine apoptosis. Representative heat density plots from two ibrutinib-resistant models demonstrate apoptosis resistance in BCWM.1/IR and RPCI-WM1/IR cells (~0–11% annexin-V positivity) relative to WT counterparts (~43–62% annexin-V positivity) after ibrutinib treatment. (**c**) Whole-exome sequencing (WES) analysis of all ibrutinib-resistant WM cell lines (*n*=3) revealed no mutation in the DNA region encoding for the Cys481 residue of the *BTK* gene as illustrated through integrative genomics viewer (IGV) visualization of nucleotide read alignments of BTK on chromosome X (Chr. X): position 100 611 161–100 611 166. (**d**) Conservation of the *BTK Cys481* region was validated by Sanger sequencing (representative chromatogram from BCWM.1/IR cells shown). (**e**) In a similar manner, WES revealed no WHIM-like mutations in the *CXCR4* gene on Chr. 2 (4 regions analyzed). One representative region shown by IGV read alignment visualization on Chr. 2, position 136 872 485, which if altered from C>A or C>G, results in a S338X mutation. (**f**) Sanger sequencing of the corresponding region region confirmed no mutation present in S338 (representative chromatogram from BCWM.1/IR cells). Additional regions corresponding to other WHIM-like mutations shown in Supplementary Figure S1.

To understand the molecular mechanisms that underlie induction of ibrutinib resistance, we exposed established human WM cell lines (*n*=3) to sequentially escalating concentrations of ibrutinib over a period of 6 months. Compared with parent WM cells (wild-type, WT), ibrutinib-resistant derivatives (denoted by IR) maintained viability and proliferative potential when exposed to lethal concentrations of ibrutinib. Median half-maximal inhibitory concentration (IC_50_) of ibrutinib-resistant clones was 22 μm (range, 20–28 μm), whereas those of WT parent cells was 3.5 μm (range, 1–6.5 μm) (Figure 1a). Resistance was also examined by means of determining apoptotic potential of ibrutinib in ibrutinib-resistant and WT WM cells. While WT cells experienced ~30–50% apoptotic cell death at concentrations ranging between 5 and 10 μm, ibrutinib-resistant clones displayed significant resistance with almost no apoptosis occurring in these cells at concentrations as high as 30 μm (Figure 1b). As *BTK^C481S^* and *CXCR4^WHIM-like^* mutations have been reported to contribute towards ibrutinib resistance,^20, 27^ we sequenced the corresponding DNA regions and found neither *BTK^C481S^* nor *CXCR4^WHIM-like^* mutation in any of the WT or ibrutinib-resistant WM cells by either whole-exome or Sanger sequencing (Figures 1c–f and Supplementary Figure S1). Altogether, these findings suggested that ibrutinib resistance in WM cells can emerge without the presence of *BTK^C481S^* or *CXCR4^WHIM-like^* mutations, indicating other potential mechanisms for WM cell survival in the presence of sustained BTK inhibition.

**Figure 2:**
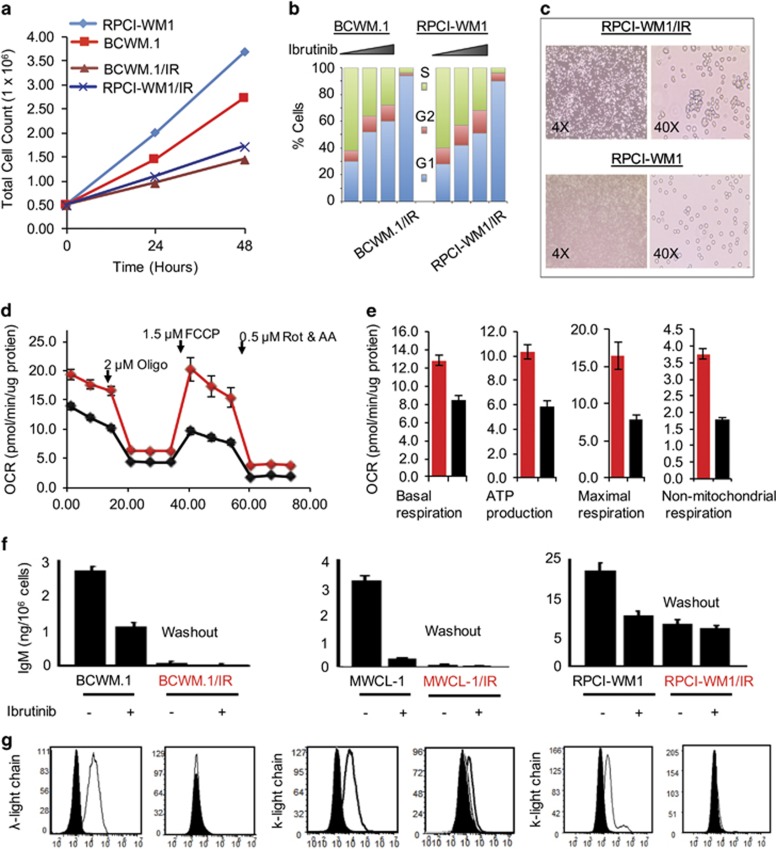
Ibrutinib-resistant WM clones show altered growth kinetics, reduced IgM secretion and Ig-light-chain expression. (**a**) WT and ibrutinib-resistant WM clones (two representative isogenic pairs shown) were seeded at 0.5 million/ml, followed by measurement of total cell numbers and viability at 24 and 48 h. (**b**) WM cells and ibrutinib-resistant derivatives (two representative isogenic pairs shown) were treated with DMSO or increasing concentrations of ibrutinib (1 or 5 μm for 24 h), followed by flow cytometry and cell cycle analysis. Ibrutinib treatment resulted in accumulation of WM tumor cells in the G1 phase of the cell cycle. By comparison, ibrutinib-resistant WM clones (BCWM.1/IR and RPCI-WM1/IR) were noted be G1 arrested. (**c**) Light microscopy showed ibrutinib-resistant WM cells to be more irregular in shape and larger in size as compared with their WT counterparts (one isogenic pair shown as representative). (**d**) Mitochondrial respiration was measured side by side in parental cells (BCWM.1) and ibrutinib-resistant WM cells (BCWM.1/IR) at baseline condition, followed by inhibition of mitochondrial ATP production (introduction of oligomycin), uncoupling oxidative phosphorylation (OP) and ATP production causing maximal mitochondrial respiration (introduction of carbonyl cyanide-4-phenylhydrazone (FCCP)) and finally complete inhibition of the OP by the combination of rotenone and antimycin A. oxygen consumption rate (OCR) is displayed in values normalized to total protein content. (**e**) Compared with parental cells, BCWM.1/IR cells showed increased values in all analyzed parameters: basal respiration (left), ATP production (middle left) and maximal respiration (middle right) and non-mitochondrial respiration (right). (**f**) IgM secretion was quantified in the supernatant of WT and ibrutinib-resistant WM clones (±ibrutinib 1μm, 24 h exposure). In WT WM cells, secretion of IgM decreased after exposure to ibrutinib. In ibrutinib-resistant clones, IgM secretion was measured in cells after a washout period (cells cultured in ibrutinib-free media for 1 week) as well as after ibrutinib treatment and was noted to be markedly reduced in washout cells compared to WT counterparts, regardless of exposure to ibrutinib thereafter. (**g**) In a similar manner, all WM cell lines were incubated with anti-λ-light-chain or anti-k-light-chain antibody and analyzed by flow cytometry, which demonstrated decreased surface expression of native Ig-light-chain on ibrutinib-resistant WM cells.

As BTK signaling is relevant to WM cell proliferation, we evaluated what effect continuous BTK suppression has on the cell cycle and proliferative potential of ibrutinib-resistant clones. Although ibrutinib-resistant WM cells continued to survive under chronic stress of ibrutinib (as these cells are kept in ibrutinib-containing media), their proliferation rate was much lower than parental WT cells. By 48 h, ibrutinib-resistant WM (BCWM.1/IR and RPCI-WM/IR) cell numbers were 1.46 and 1.73 million/ml, respectively, vs 2.73 and 3.69 million/ml in their respective WT counterparts (Figure 2a). Focused transcriptome analysis of ibrutinib-resistant cells revealed downregulation in the cell cycle genes, *CDKN1A*, *CDKN2B* in RPCI-WM1/IR vs RPCI-WM1 cells (Supplementary Figure S2 and Supplementary Table S1) and *CDKN2A* in BCWM.1/IR vs BCWM.1 cells (Supplementary Table S2). Cell cycle analysis by flow cytometry at 12 h showed that while ibrutinib treatment of WT WM cells retarded cell growth/division (as is consistent with the drugs known cytostatic effect), ibrutinib-resistant cells (after 48 h washout) remained in a growth-arrested state where >90% of cells were in the G1/G2 phase of the cell cycle (Figure 2b). Light microscopy showed that ibrutinib-resistant cells tended to cluster, and phenotypically were larger in size and less uniform in shape, confirming the inability of these cells to complete the G1 phase (Figure 2c).

The G1 phase of the cell cycle has been shown to be associated with changes in the mitochondria and increased respiration.^28^ This led us to examine mitochondrial welfare and bioenergetics, comparing mitochondrial respiration in parental and resistant cells. Using the Seahorse XF96 Flux Analyzer and examining BCWM.1 and its corresponding ibrutinib-resistant subclone (BCWM.1/IR), we found the resistant cells to have an overall increased oxygen consumption rate compared with parental cells (Figure 2d). Basal respiration in the resistant cells was increased by 30%. A similar level of increase was found in mitochondrial ATP production supporting the observation of increased mitochondrial utilization in these cells. Moreover, carbonyl cyanide-4-phenylhydrazone treatment revealed elevation in maximal respiration that reflects higher spare respiratory capacity of mitochondria in BCWM.1/IR cells as compared with parental BCWM.1 cell (Figure 2e). These findings suggest higher capability of ibrutinib-resistant cells to maintain and sustain increased mitochondrial demand under stress conditions. Moreover, we noted more than 50% increase in non-mitochondrial oxygen consumption/respiration, suggesting the ability of mitochondria of ibrutinib-resistant WM cells to adapt in a pro-oxidative environment, thus making them more resilient toward stress-associated cytoxicity.

We also examined IgM secretion from the ibrutinib-resistant cells and found it to be significantly decreased in all resistant clones (after 48 h washout and reintroduction to ibrutinib) compared with WT cells (before and after treatment with ibrutinib) (Figure 2f). Consistent with this effect, we also observed reduced cell surface expression of cognate Ig-light-chain receptors on ibrutinib-resistant WM cells (Figure 2g). Altogether, these data indicate the altered proliferative capacity yet tenacity of ibrutinib-resistant cells to maintain survival under selective pressure from ibrutinib, through mechanisms independent of *BTK^C481S^* or *CXCR4^WHIM-like^* mutations.

### BCR signaling is attenuated in ibrutinib-resistant WM cells without consequence to tumor cell viability and growth

**Figure 3:**
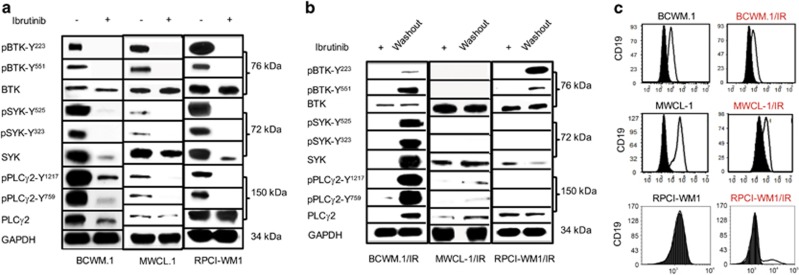
BCR signaling is dowregulated in ibrutinib-resistant WM cells. (**a**) WT WM cell lines were treated with DMSO or ibrutinib (1 μm) for 12 h and subjected to immunoblotting with anti-BTK/pBTK, anti-SYK/pSYK, anti-PLCγ2/pPLCγ2 and GAPDH antibodies. Ibrutinib treatment effectively reduced pBTK, pSYK and pPLCγ2 protein levels in all WT WM cell lines, indicating attenuation of BCR signaling. (**b**) In a similar manner, ibrutinib-resistant WM clones were also probed with anti-BTK/pBTK, anti-SYK/pSYK, anti-PLCγ2/pPLCγ2 and GAPDH antibodies. While immunobot analysis showed very low pBTK, pSYK or pPLCγ2 protein levels in ibrutinib-resistant clones (kept in ibrutinib-containing media at >90% viability), protein levels increased after washout of cells (after 48 h in ibrutinib-free media). Notably, viability of ibrutinib-resistant clones- before or after washout, was not impacted. (**c**) All WM cell lines were incubated with anti-CD19 antibody followed by flow cytometry. Compared to WT parental cells, BCWM.1/IR and MWCL-1/IR cells showed reduced surface expression of CD19. RPCI-WM1 is known to be CD19- and this was also noted in RPCI-WM1/IR cells.

As ibrutinib abrogates BTK-mediated signaling that is propagated through the B-cell receptor (BCR) hub,^5, 29^ we examined this signaling pathway in all WM cell lines. Ibrutinib treatment (1 μm) of WT WM cell lines markedly decreased phospho (p)BTK, pPLCγ2 and pSYK levels, demonstrating drug–target engagement and inhibition of BCR signaling as would be anticipated (Figure 3a). Contrastingly, in ibrutinib-resistant derivatives, we noted constitutively decreased pBTK, pPLCγ2 and pSYK protein levels—with the cells retaining >90% viability. Interestingly, after 48 h washout and culture in ibrutinib-free media, we observed marked upregulation of pBTK, pPLCγ2 and pSYK, but this did not impact ibrutinib-resistant cell viability (Figure 3b). As CD19 can complex with the BCR receptor to enhance signaling,^30^ we examined CD19 surface receptor expression by flow cytometry and observed significant decrease in ibrutinib-resistant cells compared with WT cells (Figure 3c). Taken together, these data suggested to us that ibrutinib-resistant WM cells shift survival and growth dependence away from BCR/BTK-mediated signaling and engage alternative pathway(s) to maintain viability.

### Phospho-AKT expression in ibrutinib-resistant cells is upregulated and its pharmacologic inhibition results in decreased tumor cell survival and apoptosis

**Figure 4:**
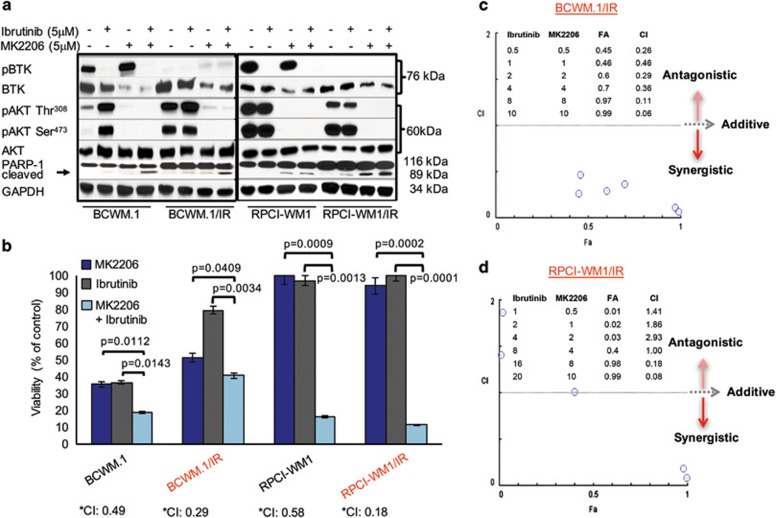
Phospho-AKT is increased in ibrutinib-resistant WM cells and its pharmacologic inhibition results in decreased tumor cell survival and apoptosis. (**a**) WM cell lines were treated with DMSO, ibrutinb or MK2206 (allosteric pan-AKT inhibitor) at indicated concentrations for 24 h and immunoblot analysis was performed probing for BTK, pBTK AKT, pAKT (Thr308), pAKT (Ser473) and PARP-1 protein expression. pAKT was noted to be decreased in all WM cells (alone or in combination with ibrutinib). PARP-1 cleavage in WM cells was most markedly observed after combination ibrutinib and MK2206 treatment. (**b**) Two isogenic cell line pairs (BCWM.1 and BCWM.1IR; RPCI-WM1 and RPCI-WM1/IR) were treated with DMSO or various concentrations of MK2206, ibrutinib or ibrutinib+MK2206 for 48 h and assessed for viability by the CellTiter Glo Luminescent Cell Assay to determine pharmacologic synergy. A combination index (CI) value of 1 signifies additivity, whereas a CI<1 signifies synergism and a CI>1 signifies antagonism between the drugs at particular concentrations. Bar graph shows representative results of combination experiments where drug combination synergy was noted. In BCWM.1 cells, ibrutinib (2 μm)+MK2206 (2 μm) reduced cell viability to 19% and in BCWM.1/IR cells, a similar trend was noted at similar concentrations, where the combination of both agents caused significant reduction in cell viability (40% viable cells) compared with either agent alone. (**c**) Representative isobologram analysis of BCWM.1/IR cells shows synergistic cytotoxic activity of ibrutinib+MK2206 in 6/6 drug combinations tested (represented by blue circles). (**d**) In RPCI-WM1 and RPCI-WM1/IR cells, ibrutinib or MK2206 alone did not significantly reduce cell viability; however, the combination of the two agents (ibrutinib 4 μm+MK2206 4 μm in RPCI-WM1 cells; ibrutinib 16 μm+MK2206 8 μm in RPCI-WM1/IR cells) significantly reduced cell viability to 16 and 11% in RPCI-WM1 and RPCI-WM1/IR cells, respectively. (**e**) Representative isobologram analysis of RPCI-WM1/IR cells showed synergistic cytotoxic activity in 2/6 combinations (represented by blue circles).

Dysfunctional AKT signaling has been reported to contribute to ibrutinib insensitivity in *CXCR4^WHIM^*+ WM cells.^9^ As such, we investigated the relevance of AKT in our *CXCR4^WHIM^*-negative ibrutinib-resistant WM cells. Targeted transcriptome analysis showed expression of genes associated with AKT signaling (such as *MET* and *WNT10B*) to be increased in RPCI-WM1/IR cells (Supplementary Table S1). Indeed, we had noted that after treatment of WT BCWM.1 cells with ibrutinib, phosphorylation of AKT (at Thr308 and Ser473) was markedly increased (Figure 4a, left panel set). And, this effect was prominent at baseline in BCWM.1/IR cells with a slight increase in pAKT Thr308 noted after 48 h washout of cells and reincubation with ibrutinib (Figure 4a, left panel set). Contrastingly, RPCI-WM1 cells, which display some growth insensitivity towards ibrutinib at baseline compared with BCWM.1 and MWCL-1 cells (Figure 1a), were noted to constitutively express pAKT at baseline, with no change in pATK following exposure to ibrutinib (Figure 4a, right panel set). Similarly, RPCI-WM1/IR cells showed constitutive expression of pAKT (more prominent at the Ser473 residue), which did not change following washout and incubation with ibrutinib (Figure 4a, right panel set). To examine functional relevance of increased pAKT in CXCR4^WHIM^-negative ibrutinib-resistant WM cells, we treated these cells (BCWM.1/IR, RPCI-WM1/IR and WT parental clones) with the clinical-grade allosteric pan-AKT inhibitor, MK2206,^31, 32^ and observed marked reduction of pAKT as well as evidence of apoptosis as indicated by PARP-1 cleavage (Figure 4a). However, when cells were treated concurrently with ibrutinib and MK2206, we noted marked reduction in pBTK and nearly complete loss of pAKT, which was associated with more robust cleavage of PARP-1 (Figure 4a). Overall, it is important to note that the minor differences noted in protein levels of different signaling molecules between the various cell line models is expected given our prior report/analysis on the variable state of generation of these cell lines (i.e. relapse/refractory vs untreated).^33, 34^ Observing that concurrent BTK and AKT inhibition could result in potentially more programmed cell death, we treated BCWM.1 and RPCI-WM1 (and their respective ibrutinib-resistant derivatives) with different concentrations of MK2206, ibrutinib or MK2206+ibrutinib for 48 h and assessed the viability. As expected, the combination of both drugs reduced WT and ibrutinib-resistant cell viability significantly more so than either ibrutinib or MK2206 alone (Figure 4b). Isobologram analysis of BCWM.1/IR cells demonstrated synergistic cytotoxic activity in 6/6 combinations (represented by blue circles) with a median combination index of 0.2 (Figure 4c). Isobologram analysis of RPCI-WM1/IR cells showed additive activity in 1/6 combinations and synergistic activity in 2/6 combinations (Figure 4d, represented by blue circles).

### Modulation of Bcl-2 expression as a function of resistance induction resulting in sensitivity to venetoclax treatment

**Figure 5:**
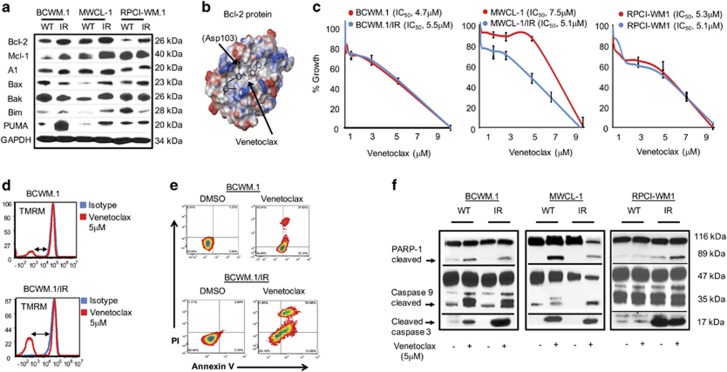
Inhibition of Bcl-2 with venetoclax results in loss of viability and mitochondrial-mediated apoptosis of ibrutinib-resistant WM cells. (**a**) WM cell lines were treated with DMSO or ibrutinib (1 μm) for 12 h and immunoblot analysis was performed probing for anti- and proapoptotic Bcl-2 family proteins. Compared with WT cells, ibrutinib-resistant clones showed increased expression of Bcl-2, with more variability in proapoptotic protein (Bax, Bak, Bim and PUMA) expression among the different ibrutinib-resistant models. (**b**) Three-dimensional (3D) rendering of venetoclax, a BH3 mimetic (black colored ligand) that selectively binds Bcl-2, occupying the P2 and P4 pockets in the BH3 groove of the Bcl-2 protein. (**c**) A 48 h MTS assay was conducted to assess sensitivity of all WM cell lines towards venetoclax, which demonstrated comparable activity in WT and ibrutinib-resistant cells alike, with the exception of MWCL-1/IR cells, which showed greater growth inhibition compared with MWCL-1 parental cells. (**d**) WM cell lines were treated with DMSO or venetoclax (5 μm) for 24 h and stained with annexin-V and propidium iodide, followed by flow cytometry to examine apoptosis. Venetoclax induced significant apoptosis in all WM cell lines tested; heat density plots from a representative isogenic pair model, BCWM.1 and BCWM.1/IR, show 60% and 63% annexin-V positivity, respectively, after treatment with the drug. (**e**) WM cells were treated with DMSO or venetoclax (5 μm) for 12 h and MOMP was measured in relation to TMRM fluorescence; TMRM-negative cells were calculated to represent (%) MOMP. MOMP was more increased in ibrutinib-resistant clones after ibrutinib exposure as compared with WT WM cells. Representative histograms are shown for BCWM.1 and BCWM.1/IR cells, where the blue line represents isotype control and the red line indicates shift in TMRM fluorescence after venetoclax treatment. (**f**) Mitochondrial-mediated apoptosis was validated in WM cells treated with DMSO or venetoclax by immunoblotting for PARP-1, caspase-9 and caspase-3 protein products, which demonstrated cleavage of these proteins in all cells treated with the drug.

In addition to compensatory AKT signaling, we investigated other potential mechanisms for ibrutinib resistance in WM cells. Deferred apoptosis is a hallmark of many B-cell malignancies associated with drug resistance;^15, 25, 35, 36^ as such, we examined expression of apoptotic regulators in our transcriptome expression data set. This analysis revealed modulation of several genes known to regulate apoptotic machinery such as *BMI1*, *GNAS*, *IL8* and *PIM1* in BCWM.1/IR and RPCI-WM1/IR cells (Supplementary Tables S1 and 2). Thus, we immunoblotted directly for apoptosis effectors; anti- (Bcl-2, Mcl-1, A1) and proapoptotic (Bax, Bak, Bim and PUMA) Bcl-2 family members in WT and ibrutinib-resistant clones and observed increased Bcl-2 and Mcl-1 in the latter, whereas proapoptotic proteins displayed more variable expression patterns (Figure 5a). This demonstrated that dysfunctional Bcl-2 interactions may have a role in maintaining ibrutinib-resistant cell survival. Next, we investigated anti-WM activity of the Bcl-2-specific BH3 mimetic, venetoclax (ABT-199, recently Food and Drug Administration-approved for treatment of 17p deletion-positive relapsed/refractory CLL),^37, 38, 39^ in WT and ibrutinib-resistant derivatives. Modeling studies of venetoclax revealed its occupancy at the P2 and P4 pockets in the BH3 groove of the Bcl-2 protein and stabilization at the Asp103 residue in particular, which is critical for specificity towards Bcl-2 (Figure 5b). WT and ibrutinib-resistant WM cells were treated with increasing concentrations of venetoclax for 48 h and cell proliferation was measured. Comparable activity of venetoclax was noted in WT and ibrutinib-resistant cells alike, with the exception of MWCL-1/IR cells, which showed greater growth inhibition compared with MWCL-1 parental cells (IC_50_ 5.1 vs 7.5 μm, respectively) (Figure 5c). Bcl-2 binds and sequesters proapoptotic BH3 proteins; interference of this dynamic results in Bax/Bak dimerization, mitochondrial pore formation and induction of apoptosis.^40^ Thus, we examined MOMP in WT and ibrutinib-resistant WM cells before and after venetoclax treatment. As anticipated, venetoclax compromised mitochondrial function in both WT and ibrutinib-resistant WM cells by increasing MOMP (Figure 5d), which was associated with induction of apoptosis as determined by annexin-V/PI staining (Figure 5e). Furthermore, (mitochondrial-mediated) programmed cell death in venetoclax-treated cells was confirmed by the presence of PARP-1 cleavage and cleaved caspase-3 (Figure 5f).

### Venetoclax synergistically enhances the antiproliferative effects of ibrutinib

**Figure 6:**
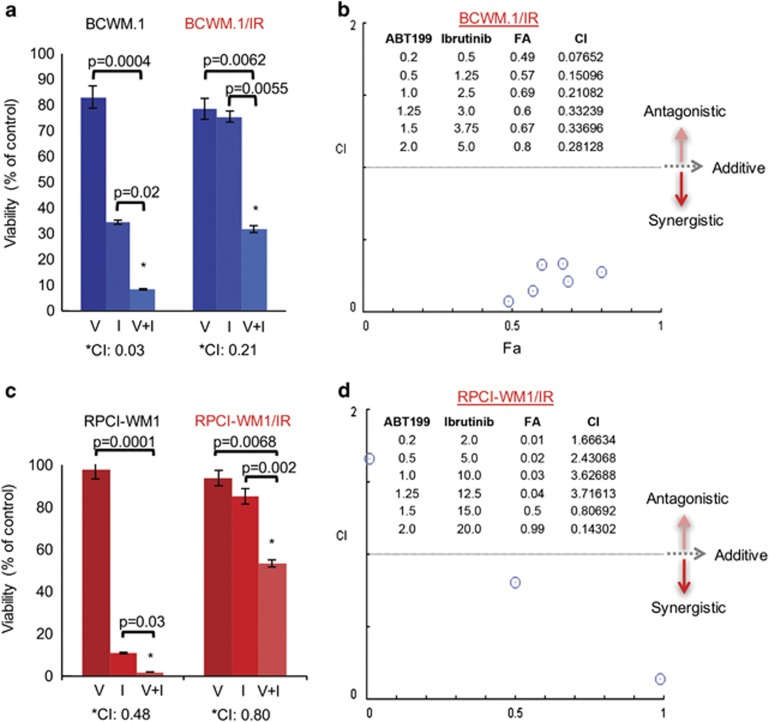
Venetoclax and ibrutinib display synergistic anti-WM activity. Two isogenic cell line pairs (BCWM.1 and BCWM.1IR; RPCI-WM1 and RPCI-WM1/IR) were treated with DMSO or various concentrations of venetoclax (V), ibrutinib (I) or venetoclax+ibrutinib (V+I) for 48 h and assessed for viability by the CellTiter Glo Luminescent Cell Assay. A combination index (CI) value of 1 signifies additivity, whereas a CI<1 signifies synergism and a CI>1 signifies antagonism between the drugs at particular concentrations. Bar graph shows representative results from combination experiments where drug combination synergy was noted. (**a**) In BCWM.1 and BCWM.1/IR cells, the combination of V (1 μm)+I (2.5 μm) synergistically reduced tumor cell viability; significantly more so than either V or I alone at a CI of 0.03 and 0.21, respectively. (**b**) Representative isobologram analysis of BCWM.1/IR cells demonstrated synergistic cytotoxic activity of V+I in 6/6 combinations (represented by blue circles). (**c**) In the RPCI-WM1 cell line and its ibrutinib-resistant derivative, the combination of V (1.5 μm)+I (15 μm) synergistically reduced tumor cell viability (CI of 0.48 and 0.80, respectively), an effect more notable than when cells were treated with V or I alone. (**d**) Representative isobologram analysis of RPCI-WM1/IR cells showed synergistic cytotoxic activity in 2/6 combinations (represented by blue circles).

Drug combination therapy produces enhanced antitumor effect and counters drug resistance.^41^ We addressed the question of whether concurrently inhibiting Bcl-2 (with venetoclax) and BTK (with ibrutinib) could induce cytotoxicity in WM cells and overcome ibrutinib-resistant WM cell insensitivity to single agent ibrutinib. Two isogenic WM cell line pairs were treated with sub-IC_50_ concentrations of venetoclax (V), ibrutinib (I) or venetoclax+ibrutinib (V+I) for 48 h and viability was assessed thereafter. In BCWM.1 and BCWM.1/IR cells, the combination of V+I synergistically reduced tumor cell viability; significantly more so than either V or I alone at a combination index of 0.03 and 0.21, respectively (Figure 6a). Isobologram analysis of BCWM.1/IR cells demonstrated synergistic cytotoxic activity of V+I in 6/6 combinations (represented by blue circles) (Figure 6b). In the RPCI-WM1 cell line and its ibrutinib-resistant derivative, the combination of V+I also synergistically reduced tumor cell viability (combination index of 0.48 and 0.80, respectively), an effect more notable than when cells were treated with V or I alone were used in ibrutinib-resistant clones (Figure 6c). Isobologram analysis of RPCI-WM1/IR cells showed that 2/6 combinations displayed synergistic cytotoxic activity (represented by blue circles) (Figure 6d). Taken in concert, our investigations reveal that altered Bcl-2 family protein expression sustains proliferation in the ibrutinib-resistant WM cells and that venetoclax induces ibrutinib-resistant cell apoptosis (see Figures 5e and f). Moreover, combination treatment of these cells with venetoclax plus ibrutinib results in enhanced loss in viability of ibrutinib-resistant cells.

## Discussion

Ibrutinib resistance represents a unique challenge due to its association with a poor clinical outcome.^12, 13^ Overall, biological understanding of how ibrutinib resistance occurs in WM remains limited. Mechanisms of resistance in other B-cell cancers are reported to be associated with ibrutinib-binding site mutations in *BTK*,^6, 8^ whereas in WM, *CXCR4^WHIM-like^* mutations have been reported to correlate with ibrutinib insensitivity in a limited number of cases.^11^ As such, the optimal therapeutic strategies to extend clinical remission in patients who have become refractory to ibrutinib are unclear and remain an active area of investigation. To this end, we dedicated our efforts towards establishment of preclinical WM models to study the phenomenon of ibrutinib resistance as is observed clinically—where chronic exposure to ibrutinib over an extended time leads to outgrowth of tumor clones that can maintain proliferative potential under ibrutinib-induced stress.

Characterization of ibrutinib-resistant clones revealed the absence of *BTK^C481S^* or *CXCR4^WHIM-like^* mutations. A C481S mutation has been shown to disrupt covalent (irreversible) binding of ibrutinib to BTK and instead permits for a reversible binding interaction, limiting the drugs effect by nearly 25-fold.^6^ The absence of a C481S anomaly in our resistant models implied that ibrutinib should optimally bind and functionally inhibit BTK, irrespective of impact on cell viability. The latter is important to note, as our assessment of phosphorylation status of BCR signalosome components in resistant cells showed that tyrosine phosphorylation of not only BTK (at Y^551^ and Y^223^ amino acids) but effectors upstream such as SYK (Y^323^ and Y^525/526^) and downstream, PLCγ2 (Y^759^ and Y^1217^), were inhibited by ibrutinib, indicating that drug binding is intact and BCR signaling is abrogated. And, this effect (BCR signalosome phosphorylation status) was reversed when resistant cells were washed out and grown in ibrutinib-free media. In either condition, ibrutinib-resistant cells remained viable with stable growth kinetics, indicating to us that in these cells BTK-mediated signaling was not a primary driver of disease. We also noted decreased IgM secretion in the sera of ibrutinib-resistant cells. It would appear that in this drug-resistant state, the emphasis of the tumor cells is more towards survivability and transitioning to an ‘oligoseceretory phase’. Clinically this is noted also in patients with plasma cell malignancies, whereby with increasing resistance to therapeutics the cell can eventually evolve into a non-secretory/oligosecretory phase.

In addition to *BTK*, we also probed for *CXCR4^WHIM-like^* mutations, which in WM have been shown to decrease CXCR4 receptor internalization, thereby allowing continuous signaling and downstream activation of ERK and AKT, leading to sustained WM cell proliferation/survival.^4^ However, the role of AKT and its relevance in WT *CXCR4* ibrutinib-resistant WM is unclear. While pAKT was effectively downregulated with MK2206 treatment alone in all WM cells tested herein, this did not translate to a significant loss of viability in WT or ibrutinib-resistant RPCI-WM1/IR cells—and could potentially just be a function of increased basal AKT activity in these cells. However, combined BTK and AKT inhibition induced a significant decrease in RPCI-WM1 and RPCI-WM1/IR cell viability, suggesting that the protective role of AKT could be overcome with concurrent targeting of BTK with ibrutinib (at sub-IC_50_ concentrations). Further studies are needed to explicate the role of AKT in *CXCR4^WHIM-like^*-negative ibrutinib-resistant WM and novel, clinically relevant inhibitors that target upstream of AKT, that is, PI3K isoforms (idelalisib, duvelesib) may hold greater anti-WM potential when combined with ibrutinib.

In conjunction with growth-stimulating signaling pathways such as those propagated by AKT/ERK/NFKB activation,^42^ WM cell survival is intricately supported by malfunctions in initiation of apoptosis through dysregulated expression of Bcl-2 family proteins.^15, 25, 43^ Interestingly, AKT and Bcl-2 are directly linked, where studies by Pugazhenthi *et al.*^44^ have shown that AKT activates the transcription factor CREB (cAMP response element-binding protein), which binds to the *Bcl-2* promoter region and positively regulates its expression. Whether upregulation of Bcl-2 in ibrutinib-resistant WM is related to CREB and AKT is currently being examined by us. Nevertheless, we have previously shown the utility of disrupting proapoptotic Bcl-2 protein interactions in CLL, WM and multiple myeloma and that this functional interference results in malignant cell death or resensitization (or heightened sensitivity) of tumor cells towards other therapeutics.^15, 25, 35, 40, 45, 46, 47, 48, 49, 50, 51^ Indeed, this observation also held true for *CXCR4^WHIM-like^*-negative ibrutinib-resistant WM cells, where the Bcl-2-specific BH3 mimetic, venetoclax, decreased ibrutinib-resistant WM cell viability and induced apoptosis as a single agent. Our investigations further highlighted that concomitant disruption of BTK and Bcl-2 with sub-IC_50_ doses of ibrutinib and venetoclax, respectively, synergistically induce tumor cell lethality in WT and ibrutinib-resistant cells. These data are in line with observations by Cao *et al.*^43^ in WM, whereas we are the first to demonstrate the utility of combined Bcl-2 and BTK inhibition in ibrutinib-resistant WM cells in which neither *BTK^C481S^* nor *CXCR4^WHIM-like^* mutations are responsible for drug resistance. The rationale for Bcl-2 plus BTK inhibition is further supported by preclinical studies in CLL^52^ and MCL;^53^ the latter where the combination of venetoclax and ibrutinib is being investigated in two separate phase clinical trials (NCT02419560, phase I study; NCT02471391, phase II study).

In conclusion, we report on the development of novel ibrutinib-resistant WM cell lines, which exhibit decreased survival dependency on BTK-mediated signaling. These models all lack ibrutinib-binding site (*BTK Cys481*) mutations as well as *CXCR4^WHIM-like^* mutations, providing new insight into mechanism underpinning ibrutinib resistance independent of these aberrations. Focused transcriptome analysis combined with protein profiling revealed AKT and Bcl-2-associated pathways may have a role in maintaining survival of ibrutinib-resistant WM cells. *In vitro* efficacy and mechanistic analysis confirmed reliance on both pathways and suggests that drug combination strategies encompassing BTK+AKT/PI3K inhibition or BTK+Bcl-2 inhibition can potentially overcome ibrutinib resistance in WM. Finally, our observations are unique as they feature previously unknown mechanisms directing resistance to ibrutinib in WM cells and provide rationale for potential combination therapeutic strategies to address this clinical problem in patients with WM.

## References

[bib1] Owen RG, Treon SP, Al-Katib A, Fonseca R, Greipp PR, McMaster ML et al. Clinicopathological definition of Waldenstrom's macroglobulinemia: consensus panel recommendations from the Second International Workshop on Waldenstrom's macroglobulinemia. Semin Oncol 2003; 30: 110–115. 12720118 10.1053/sonc.2003.50082

[bib2] Oza A, Rajkumar SV. Waldenstrom macroglobulinemia: prognosis and management. Blood Cancer J 2015; 5: e296. 25815903 10.1038/bcj.2015.28 PMC4382666

[bib3] Treon SP, Tripsas CK, Meid K, Warren D, Varma G, Green R et al. Ibrutinib in previously treated Waldenstrom's macroglobulinemia. N Engl J Med 2015; 372: 1430–1440. 25853747 10.1056/NEJMoa1501548

[bib4] Cao Y, Hunter ZR, Liu X, Xu L, Yang G, Chen J et al. The WHIM-like CXCR4(S338X) somatic mutation activates AKT and ERK, and promotes resistance to ibrutinib and other agents used in the treatment of Waldenstrom's macroglobulinemia. Leukemia 2015; 29: 169–176. 24912431 10.1038/leu.2014.187

[bib5] Young RM, Staudt LM. Ibrutinib treatment of CLL: the cancer fights back. Cancer Cell 2014; 26: 11–13. 25026208 10.1016/j.ccr.2014.06.023 PMC4199743

[bib6] Woyach JA, Furman RR, Liu TM, Ozer HG, Zapatka M, Ruppert AS et al. Resistance mechanisms for the Bruton's tyrosine kinase inhibitor ibrutinib. N Engl J Med 2014; 370: 2286–2294. 24869598 10.1056/NEJMoa1400029 PMC4144824

[bib7] Furman RR, Cheng S, Lu P, Setty M, Perez AR, Guo A et al. Ibrutinib resistance in chronic lymphocytic leukemia. N Engl J Med 2014; 370: 2352–2354. 24869597 10.1056/NEJMc1402716 PMC4512173

[bib8] Chiron D, Di Liberto M, Martin P, Huang X, Sharman J, Blecua P et al. Cell-cycle reprogramming for PI3K inhibition overrides a relapse-specific C481S BTK mutation revealed by longitudinal functional genomics in mantle cell lymphoma. Cancer Discov 2014; 4: 1022–1035. 25082755 10.1158/2159-8290.CD-14-0098 PMC4155003

[bib9] Cao Y, Hunter ZR, Liu X, Xu L, Yang G, Chen J et al. CXCR4 WHIM-like frameshift and nonsense mutations promote ibrutinib resistance but do not supplant MYD88-directed survival signalling in Waldenstrom macroglobulinaemia cells. Br J Haematol 2014 2015; 168: 701–707. 10.1111/bjh.13200 25371371

[bib10] Treon SP, Cao Y, Xu L, Yang G, Liu X, Hunter ZR. Somatic mutations in MYD88 and CXCR4 are determinants of clinical presentation and overall survival in Waldenstrom macroglobulinemia. Blood 2014; 123: 2791–2796. 24553177 10.1182/blood-2014-01-550905

[bib11] Treon SP, Xu L, Hunter Z. MYD88 mutations and response to ibrutinib in Waldenstrom's macroglobulinemia. N Engl J Med 2015; 373: 584–586. 10.1056/NEJMc1506192 26244327

[bib12] Jain P, Keating M, Wierda W, Estrov Z, Ferrajoli A, Jain N et al. Outcomes of patients with chronic lymphocytic leukemia after discontinuing ibrutinib. Blood 2015; 125: 2062–2067. 25573991 10.1182/blood-2014-09-603670 PMC4467871

[bib13] Martin P, Maddocks K, Leonard JP, Ruan J, Goy A, Wagner-Johnston N et al. Post-ibrutinib outcomes in patients with mantle cell lymphoma. Blood 2016; 127: 1559–1563. 26764355 10.1182/blood-2015-10-673145

[bib14] Chitta KS, Paulus A, Kuranz-Blake M, Akhtar S, Novak AJ, Ansell SM et al. Acquired *in vitro* resistance to ibrutinib is associated with transcriptional re-programming and sustained survival signaling in Waldenströms macroglobulinemia and mantle cell lymphoma, independent of BTK Cys481 mutation. Blood 2014; 124: 2250–2250 (abstract 2250).

[bib15] Chitta K, Paulus A, Caulfield TR, Akhtar S, Blake MK, Ailawadhi S et al. Nimbolide targets BCL2 and induces apoptosis in preclinical models of Waldenstroms macroglobulinemia. Blood Cancer J 2014; 4: e260. 25382610 10.1038/bcj.2014.74 PMC5424099

[bib16] Paulus A, Akhtar S, Caulfield TR, Samuel K, Yousaf H, Bashir Y et al. Coinhibition of the deubiquitinating enzymes, USP14 and UCHL5, with VLX1570 is lethal to ibrutinib- or bortezomib-resistant Waldenstrom macroglobulinemia tumor cells. Blood Cancer J 2016; 6: e492. 27813535 10.1038/bcj.2016.93 PMC5148058

[bib17] Honigberg LA, Smith AM, Sirisawad M, Verner E, Loury D, Chang B et al. The Bruton tyrosine kinase inhibitor PCI-32765 blocks B-cell activation and is efficacious in models of autoimmune disease and B-cell malignancy. Proc Natl Acad Sci USA 2010; 107: 13075–13080. 20615965 10.1073/pnas.1004594107 PMC2919935

[bib18] Cheng S, Guo A, Lu P, Ma J, Coleman M, Wang YL. Functional characterization of BTK(C481S) mutation that confers ibrutinib resistance: exploration of alternative kinase inhibitors. Leukemia 2015; 29: 895–900. 25189416 10.1038/leu.2014.263

[bib19] Chang BY, Furman RR, Zapatka M, Barrientos JC, Li D, Steggerda S et al. Poster discussion session, Leukemia, Myelodysplasia, and transplantation. J Clin Oncol 2013; 31(suppl): abstr 7014.

[bib20] Zhang SQ, Smith SM, Zhang SY, Lynn Wang Y. Mechanisms of ibrutinib resistance in chronic lymphocytic leukaemia and non-Hodgkin lymphoma. Br J Haematol 2015; 170: 445–456. 25858358 10.1111/bjh.13427

[bib21] Paulus A, Ailawadhi S, Chanan-Khan A. Novel therapeutic targets in Waldenstrom macroglobulinemia. Best Pract Res Clin Haematol 2016; 29: 216–228. 27825468 10.1016/j.beha.2016.08.020

[bib22] Chitta K, Paulus A, Akhtar S, Blake MK, Caulfield TR, Novak AJ et al. Targeted inhibition of the deubiquitinating enzymes, USP14 and UCHL5, induces proteotoxic stress and apoptosis in Waldenstrom macroglobulinaemia tumour cells. Br J Haematol 2015; 169: 377–390. 25691154 10.1111/bjh.13304 PMC4846423

[bib23] Chou TC, Talalay P. Quantitative analysis of dose–effect relationships: the combined effects of multiple drugs or enzyme inhibitors. Adv Enzyme Regul 1984; 22: 27–55. 6382953 10.1016/0065-2571(84)90007-4

[bib24] Chitta KS, Khan AN, Ersing N, Swaika A, Masood A, Paulus A et al. Neem leaf extract induces cell death by apoptosis and autophagy in B-chronic lymphocytic leukemia cells. Leuk Lymphoma 2014; 55: 652–661. 23721511 10.3109/10428194.2013.807927

[bib25] Paulus A, Chitta K, Akhtar S, Personett D, Miller KC, Thompson KJ et al. AT-101 downregulates BCL2 and MCL1 and potentiates the cytotoxic effects of lenalidomide and dexamethasone in preclinical models of multiple myeloma and Waldenstrom macroglobulinaemia. Br J Haematol 2014; 164: 352–365. 24236538 10.1111/bjh.12633 PMC4406280

[bib26] Geiss GK, Bumgarner RE, Birditt B, Dahl T, Dowidar N, Dunaway DL et al. Direct multiplexed measurement of gene expression with color-coded probe pairs. Nat Biotechnol 2008; 26: 317–325. 18278033 10.1038/nbt1385

[bib27] Hunter ZR, Xu L, Yang G, Zhou Y, Liu X, Cao Y et al. The genomic landscape of Waldenstrom macroglobulinemia is characterized by highly recurring MYD88 and WHIM-like CXCR4 mutations, and small somatic deletions associated with B-cell lymphomagenesis. Blood 2014; 123: 1637–1646. 24366360 10.1182/blood-2013-09-525808

[bib28] Schieke SM, McCoy JP Jr, Finkel T. Coordination of mitochondrial bioenergetics with G1 phase cell cycle progression. Cell Cycle 2008; 7: 1782–1787. 18583942 10.4161/cc.7.12.6067 PMC3399174

[bib29] Oppezzo P, Dighiero G. 'Role of the B-cell receptor and the microenvironment in chronic lymphocytic leukemia'. Blood Cancer J 2013; 3: e149. 24056719 10.1038/bcj.2013.45 PMC3789209

[bib30] Depoil D, Fleire S, Treanor BL, Weber M, Harwood NE, Marchbank KL et al. CD19 is essential for B cell activation by promoting B cell receptor-antigen microcluster formation in response to membrane-bound ligand. Nat Immunol 2008; 9: 63–72. 18059271 10.1038/ni1547

[bib31] Yap TA, Yan L, Patnaik A, Tunariu N, Biondo A, Fearen I et al. Interrogating two schedules of the AKT inhibitor MK-2206 in patients with advanced solid tumors incorporating novel pharmacodynamic and functional imaging biomarkers. Clin Cancer Res 2014; 20: 5672–5685. 25239610 10.1158/1078-0432.CCR-14-0868 PMC4233149

[bib32] Lopez-Chavez A, Thomas A, Rajan A, Raffeld M, Morrow B, Kelly R et al. Molecular profiling and targeted therapy for advanced thoracic malignancies: a biomarker-derived, multiarm, multihistology phase II basket trial. J Clin Oncol 2015; 33: 1000–1007. 25667274 10.1200/JCO.2014.58.2007 PMC4356709

[bib33] Paulus A, Chitta KS, Wallace PK, Advani PP, Akhtar S, Kuranz-Blake M et al. Immunophenotyping of Waldenstroms macroglobulinemia cell lines reveals distinct patterns of surface antigen expression: potential biological and therapeutic implications. PLoS ONE 2015; 10: e0122338. 25853860 10.1371/journal.pone.0122338 PMC4390194

[bib34] Ailawadhi S, Paulus A, Chanan-Khan A. Preclinical models of Waldenstrom's macroglobulinemia and drug resistance. Best Pract Res Clin Haematol 2016; 29: 169–178. 27825463 10.1016/j.beha.2016.08.017

[bib35] Chanan-Khan A. Bcl-2 antisense therapy in B-cell malignancies. Blood Rev 2005; 19: 213–221. 15784299 10.1016/j.blre.2004.11.002

[bib36] Chanan-Khan A. Bcl-2 antisense therapy in hematologic malignancies. Curr Opin Oncol 2004; 16: 581–585. 15627020 10.1097/01.cco.0000142074.67968.eb

[bib37] Roberts AW, Davids MS, Pagel JM, Kahl BS, Puvvada SD, Gerecitano JF et al. Targeting BCL2 with venetoclax in relapsed chronic lymphocytic leukemia. N Engl J Med 2016; 374: 311–322. 26639348 10.1056/NEJMoa1513257 PMC7107002

[bib38] Souers AJ, Leverson JD, Boghaert ER, Ackler SL, Catron ND, Chen J et al. ABT-199, a potent and selective BCL-2 inhibitor, achieves antitumor activity while sparing platelets. Nat Med 2013; 19: 202–208. 23291630 10.1038/nm.3048

[bib39] Phillips DC, Xiao Y, Lam LT, Litvinovich E, Roberts-Rapp L, Souers AJ et al. Loss in MCL-1 function sensitizes non-Hodgkin's lymphoma cell lines to the BCL-2-selective inhibitor venetoclax (ABT-199). Blood Cancer J 2015; 5: e368. 26565405 10.1038/bcj.2015.88 PMC4670945

[bib40] Patel MP, Masood A, Patel PS, Chanan-Khan AA. Targeting the Bcl-2. Curr Opin Oncol 2009; 21: 516–523. 19730103 10.1097/CCO.0b013e328331a7a4

[bib41] Szakacs G, Paterson JK, Ludwig JA, Booth-Genthe C, Gottesman MM. Targeting multidrug resistance in cancer. Nat Rev Drug Discov 2006; 5: 219–234. 16518375 10.1038/nrd1984

[bib42] Ansell SM, Hodge LS, Secreto FJ, Manske M, Braggio E, Price-Troska T et al. Activation of TAK1 by MYD88 L265P drives malignant B-cell growth in non-Hodgkin lymphoma. Blood Cancer J 2014; 4: e183. 24531446 10.1038/bcj.2014.4 PMC3944662

[bib43] Cao Y, Yang G, Hunter ZR, Liu X, Xu L, Chen J et al. The BCL2 antagonist ABT-199 triggers apoptosis, and augments ibrutinib and idelalisib mediated cytotoxicity in CXCR4 wild-type and CXCR4 WHIM mutated Waldenstrom macroglobulinaemia cells. Br J Haematol 2015; 170: 134–138. 25582069 10.1111/bjh.13278

[bib44] Pugazhenthi S, Nesterova A, Sable C, Heidenreich KA, Boxer LM, Heasley LE et al. Akt/protein kinase B up-regulates Bcl-2 expression through cAMP-response element-binding protein. J Biol Chem 2000; 275: 10761–10766. 10753867 10.1074/jbc.275.15.10761

[bib45] Chanan-Khan A, Czuczman MS. Bcl-2 antisense therapy in B-cell malignant proliferative disorders. Curr Treat Option Oncol 2004; 5: 261–267. 10.1007/s11864-004-0017-3 15233903

[bib46] Chanan-Khan AA. Bcl-2 antisense therapy in multiple myeloma. Oncology 2004; 18(Suppl 10): 21–24. 15651173

[bib47] Masood A, Chitta K, Paulus A, Khan AN, Sher T, Ersing N et al. Downregulation of BCL2 by AT-101 enhances the antileukaemic effect of lenalidomide both by an immune dependant and independent manner. Br J Haematol 2012; 157: 59–66. 22171982 10.1111/j.1365-2141.2011.08984.x

[bib48] Advani PP, Paulus A, Masood A, Sher T, Chanan-Khan A. Pharmacokinetic evaluation of oblimersen sodium for the treatment of chronic lymphocytic leukemia. Expert Opin Drug Metab Toxicol 2011; 7: 765–774. 21521129 10.1517/17425255.2011.579105

[bib49] Chanan-Khan AA, Niesvizky R, Hohl RJ, Zimmerman TM, Christiansen NP, Schiller GJ et al. Phase III randomised study of dexamethasone with or without oblimersen sodium for patients with advanced multiple myeloma. Leuk Lymphoma 2009; 50: 559–565. 19373653 10.1080/10428190902748971

[bib50] Ramanarayanan J, Hernandez-Ilizaliturri FJ, Chanan-Khan A, Czuczman MS. Pro-apoptotic therapy with the oligonucleotide Genasense (oblimersen sodium) targeting Bcl-2 protein expression enhances the biological anti-tumour activity of rituximab. Br J Haematol 2004; 127: 519–530. 15566355 10.1111/j.1365-2141.2004.05239.x

[bib51] O'Brien S, Moore JO, Boyd TE, Larratt LM, Skotnicki A, Koziner B et al. Randomized phase III trial of fludarabine plus cyclophosphamide with or without oblimersen sodium (Bcl-2 antisense) in patients with relapsed or refractory chronic lymphocytic leukemia. J Clin Oncol 2007; 25: 1114–1120. 17296974 10.1200/JCO.2006.07.1191

[bib52] Cervantes-Gomez F, Lamothe B, Woyach JA, Wierda WG, Keating MJ, Balakrishnan K et al. Pharmacological and protein profiling suggests venetoclax (ABT-199) as optimal partner with ibrutinib in chronic lymphocytic leukemia. Clin Cancer Res 2015; 21: 3705–3715. 25829398 10.1158/1078-0432.CCR-14-2809 PMC4537801

[bib53] Zhao X, Bodo J, Sun D, Durkin L, Lin J, Smith MR et al. Combination of ibrutinib with ABT-199: synergistic effects on proliferation inhibition and apoptosis in mantle cell lymphoma cells through perturbation of BTK, AKT and BCL2 pathways. Br J Haematol 2015; 168: 765–768. 25284608 10.1111/bjh.13149

