# Supplementary figures and images for: Waldenstrom macroglobulinemia cells devoid of BTKC481S or CXCR4WHIM-like mutations acquire resistance to ibrutinib through upregulation of Bcl-2 and AKT resulting in vulnerability towards venetoclax or MK2206 treatment
Source: Blood Cancer J. 2017 May 26;7(5):e565–. doi: 10.1038/bcj.2017.40 (PMC5518884; doi:10.1038/bcj.2017.40)

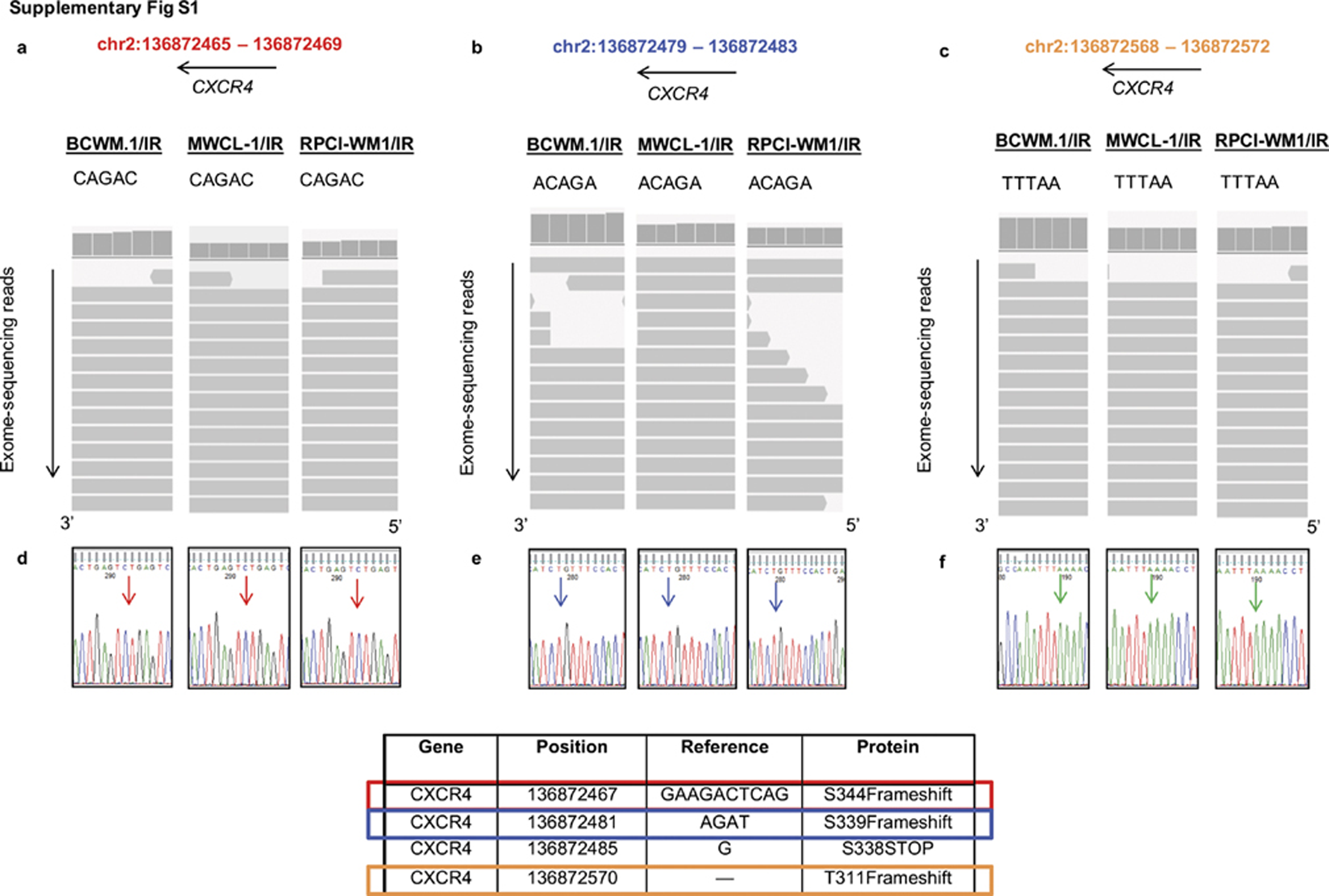

Supplement: Supplementary Figure 1 [file bcj201740x1.tif]

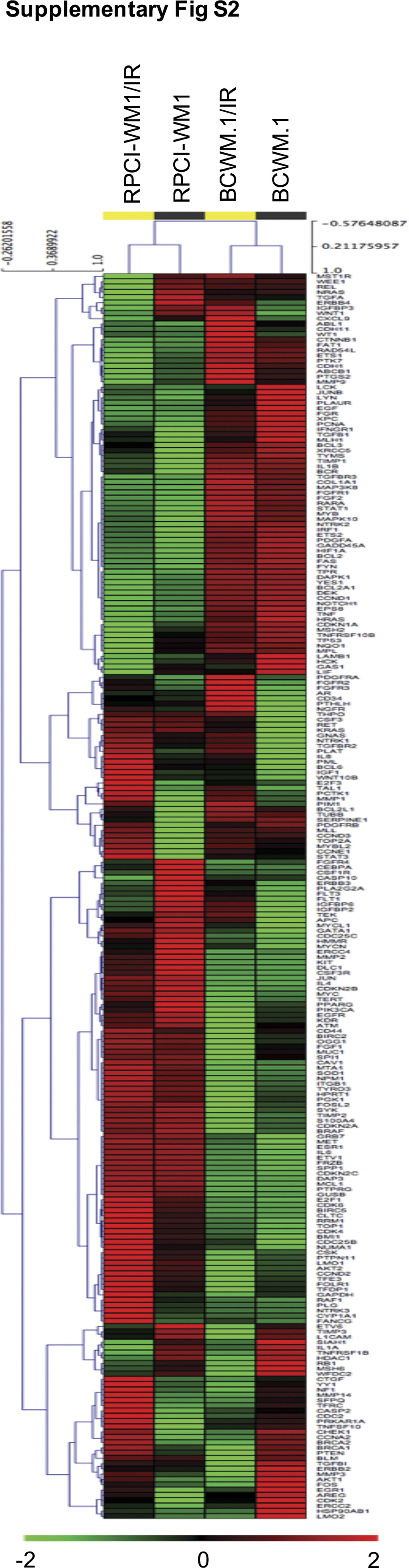

Supplement: Supplementary Figure 2 [file bcj201740x2.tif]
