# Supplementary material for: Waldenstrom macroglobulinemia cells devoid of BTKC481S or CXCR4WHIM-like mutations acquire resistance to ibrutinib through upregulation of Bcl-2 and AKT resulting in vulnerability towards venetoclax or MK2206 treatment
Source: Blood Cancer J. 2017 May 26;7(5):e565–. doi: 10.1038/bcj.2017.40 (PMC5518884; doi:10.1038/bcj.2017.40)
Supplement: Supplementary Table 1 [file bcj201740x3.docx]

**Supplementary Table 1.**

**Differential expression of genes in RPCI-WM1/IR cells vs. RPCI-WM1 cells (NanoString nCounter targeted transcriptome analysis)**

| **Gene** | **RPCI-WM/IR** | **RPCI-WM1** | **fold change** |
| --- | --- | --- | --- |
| ERBB3 | 1.28 | 12.38 | -9.671875 |
| CXCL9 | 1.28 | 8.85 | -6.9140625 |
| TNFRSF10B | 7.69 | 52.19 | -6.786736021 |
| TGFA | 1.28 | 7.96 | -6.21875 |
| ERBB4 | 2.56 | 7.96 | -3.109375 |
| MYCL1 | 7.69 | 23.88 | -3.105331599 |
| CDKN1A | 43.57 | 130.91 | -3.004590314 |
| CDKN2B | 35.88 | 106.14 | -2.95819398 |
| REL | 26.91 | 76.95 | -2.859531773 |
| TNF | 3.84 | 10.61 | -2.763020833 |
| FGFR4 | 5.13 | 10.61 | -2.068226121 |
| TNFRSF1B | 144.8 | 276.86 | -1.912016575 |
| YES1 | 6.41 | 11.5 | -1.794071763 |
| SIAH1 | 7.69 | 13.27 | -1.725617685 |
| NRAS | 107.64 | 171.6 | -1.594202899 |
| ATM | 8.97 | 14.15 | -1.577480491 |
| NQO1 | 222.97 | 337.89 | -1.51540566 |
| SPP1 | 5025.79 | 7588.33 | -1.509878049 |
| MYCN | 7.69 | 11.5 | -1.495448635 |
| PIK3CA | 201.19 | 297.2 | -1.477210597 |
| ETS1 | 248.6 | 363.54 | -1.462349155 |
| TP53 | 347.27 | 498.87 | -1.436547931 |
| DEK | 274.23 | 387.42 | -1.412755716 |
| MYC | 1906.78 | 2590.77 | -1.358714692 |
| EPS8 | 32.04 | 42.46 | -1.325218477 |
| NOTCH1 | 46.13 | 61.03 | -1.323000217 |
| TERT | 29.47 | 38.92 | -1.320665083 |
| HDAC1 | 1144.32 | 1509 | -1.318687081 |
| TIMP2 | 158.9 | 208.75 | -1.31371932 |
| ERCC4 | 46.13 | 59.26 | -1.284630392 |
| HMMR | 237.07 | 298.08 | -1.25735015 |
| JUN | 64.07 | 79.61 | -1.242547214 |
| CDKN2A | 52.54 | 64.57 | -1.228968405 |
| FAT1 | 11.53 | 14.15 | -1.227233304 |
| APC | 28.19 | 33.61 | -1.192266761 |
| MSH2 | 503.6 | 597.94 | -1.187331215 |
| ESR1 | 30.75 | 36.27 | -1.179512195 |
| SYK | 693.26 | 790.76 | -1.140639875 |
| ETV6 | 618.93 | 696.12 | -1.124715234 |
| SPI1 | 278.07 | 312.24 | -1.122882727 |
| BRAF | 242.19 | 271.55 | -1.121227136 |
| RB1 | 324.2 | 351.16 | -1.083158544 |
| CDC25C | 47.41 | 51.3 | -1.0820502 |
| HRAS | 115.33 | 123.83 | -1.073701552 |
| RAD54L | 167.87 | 176.02 | -1.048549473 |
| S100A4 | 2993.44 | 3134.75 | -1.047206558 |
| TGFBR3 | 7.69 | 7.96 | -1.035110533 |
| CTNNB1 | 750.92 | 771.31 | -1.027153359 |
| MSH6 | 299.86 | 307.81 | -1.026512372 |
| TPR | 275.51 | 278.63 | -1.011324453 |
| ETV1 | 89.7 | 90.22 | -1.005797101 |
| IL6 | 74.32 | 74.3 | 1.000269179 |
| KRAS | 461.32 | 460.84 | 1.001041576 |
| FOSL2 | 62.79 | 61.92 | 1.014050388 |
| MCL1 | 1837.58 | 1810.62 | 1.014889927 |
| BIRC2 | 354.96 | 344.96 | 1.028988868 |
| LYN | 166.59 | 160.98 | 1.03484905 |
| ERBB2 | 6.41 | 6.19 | 1.035541195 |
| GRB7 | 6.41 | 6.19 | 1.035541195 |
| DAP3 | 511.29 | 493.56 | 1.035922684 |
| OGG1 | 153.77 | 147.72 | 1.040955862 |
| ABL1 | 222.97 | 213.17 | 1.045972698 |
| XPC | 112.77 | 107.03 | 1.053629823 |
| BLM | 174.28 | 164.52 | 1.059324094 |
| CD44 | 982.86 | 919.02 | 1.0694653 |
| HSP90AB1 | 8795.78 | 8152.66 | 1.078884683 |
| WT1 | 3.84 | 3.54 | 1.084745763 |
| THPO | 7.69 | 7.08 | 1.086158192 |
| RRM1 | 831.65 | 754.5 | 1.102253148 |
| SFPQ | 2564.15 | 2313.03 | 1.10856755 |
| HPRT1 | 2397.57 | 2145.86 | 1.11730029 |
| NPM1 | 56.38 | 50.42 | 1.118207061 |
| BCL2 | 99.95 | 89.34 | 1.118759794 |
| SOD1 | 211.44 | 188.4 | 1.122292994 |
| NUMA1 | 221.69 | 195.48 | 1.134080213 |
| HIF1A | 471.57 | 413.96 | 1.139168036 |
| JUNB | 101.23 | 88.45 | 1.144488412 |
| AKT2 | 780.39 | 676.66 | 1.153297077 |
| PCNA | 547.17 | 474.11 | 1.15409926 |
| CSF3 | 5.13 | 4.42 | 1.160633484 |
| FGF1 | 5.13 | 4.42 | 1.160633484 |
| FGFR3 | 5.13 | 4.42 | 1.160633484 |
| PLAT | 5.13 | 4.42 | 1.160633484 |
| TGFBI | 5.13 | 4.42 | 1.160633484 |
| CLTC | 879.07 | 757.15 | 1.161024896 |
| PGK1 | 6238.03 | 5353.14 | 1.165302981 |
| GUSB | 253.72 | 217.59 | 1.166046234 |
| MLH1 | 298.57 | 254.74 | 1.172057784 |
| GADD45A | 217.84 | 183.98 | 1.184041744 |
| ERCC2 | 116.61 | 98.18 | 1.187716439 |
| STAT3 | 502.32 | 422.8 | 1.18807947 |
| FOLR1 | 6.41 | 5.31 | 1.207156309 |
| MAP3K8 | 6.41 | 5.31 | 1.207156309 |
| RARA | 19.22 | 15.92 | 1.207286432 |
| IGF1 | 20.5 | 16.81 | 1.219512195 |
| BIRC5 | 1619.74 | 1321.48 | 1.225701486 |
| TFDP1 | 1456.99 | 1184.38 | 1.23017106 |
| CDKN2C | 1335.26 | 1074.7 | 1.242449056 |
| TFE3 | 16.66 | 13.27 | 1.255463451 |
| CDK6 | 566.39 | 444.03 | 1.275566966 |
| TFRC | 2814.03 | 2205.12 | 1.276134632 |
| IRF1 | 102.51 | 79.61 | 1.287652305 |
| IFNGR1 | 189.65 | 146.83 | 1.291629776 |
| PTPN11 | 730.42 | 565.21 | 1.292298438 |
| CCND2 | 3912.23 | 3020.65 | 1.295161637 |
| TUBB | 2917.83 | 2252 | 1.295661634 |
| CCNA2 | 1679.96 | 1289.64 | 1.302658106 |
| TYRO3 | 11.53 | 8.85 | 1.302824859 |
| CSK | 1631.27 | 1244.53 | 1.31075185 |
| YY1 | 977.74 | 745.65 | 1.311258633 |
| MYB | 187.09 | 142.41 | 1.313742012 |
| CDK4 | 720.17 | 544.87 | 1.321728119 |
| CHEK1 | 759.89 | 573.17 | 1.325767224 |
| E2F1 | 330.61 | 247.67 | 1.334881092 |
| FRZB | 4850.24 | 3629.2 | 1.336448804 |
| MLL | 110.2 | 82.26 | 1.339654753 |
| BRCA1 | 239.63 | 178.67 | 1.341187664 |
| CDK2 | 162.74 | 121.18 | 1.342960885 |
| PRKAR1A | 1551.82 | 1154.3 | 1.344381876 |
| PTPRG | 79.45 | 58.38 | 1.360911271 |
| XRCC5 | 5220.57 | 3829.99 | 1.363076666 |
| RAF1 | 799.62 | 584.67 | 1.367643286 |
| MTA1 | 3787.93 | 2765.91 | 1.369505877 |
| TYMS | 3266.38 | 2368.76 | 1.37894088 |
| TOP1 | 2758.93 | 1996.37 | 1.381973282 |
| CDC2 | 1843.99 | 1331.21 | 1.385198428 |
| CAV1 | 271.66 | 195.48 | 1.389707387 |
| BCR | 296.01 | 212.29 | 1.394366197 |
| MET | 92.26 | 65.45 | 1.409625668 |
| BRCA2 | 247.32 | 175.14 | 1.412127441 |
| PTEN | 1127.66 | 793.42 | 1.421264904 |
| PCTK1 | 329.33 | 229.98 | 1.431994086 |
| PDGFRA | 7.69 | 5.31 | 1.448210923 |
| NTRK1 | 5.13 | 3.54 | 1.449152542 |
| IL8 | 6.41 | 4.42 | 1.450226244 |
| TGFB1 | 362.65 | 247.67 | 1.46424678 |
| STAT1 | 709.92 | 483.83 | 1.467292231 |
| PML | 56.38 | 38.03 | 1.482513805 |
| NF1 | 280.63 | 185.75 | 1.510794078 |
| TOP2A | 918.79 | 607.67 | 1.511988415 |
| FANCG | 240.91 | 157.45 | 1.530073039 |
| GAPDH | 30154.77 | 19281.75 | 1.563902135 |
| CCNE1 | 249.88 | 159.21 | 1.569499403 |
| WNT10B | 15.38 | 9.73 | 1.580678314 |
| FOS | 12.81 | 7.96 | 1.609296482 |
| CDC25B | 352.4 | 214.05 | 1.646344312 |
| CCND3 | 92.26 | 55.73 | 1.655481787 |
| BMI1 | 1002.08 | 578.48 | 1.732263864 |
| MYBL2 | 2265.58 | 1293.17 | 1.751958366 |
| CASP2 | 497.2 | 281.28 | 1.767633675 |
| LMO1 | 6.41 | 3.54 | 1.810734463 |
| PDGFA | 6.41 | 3.54 | 1.810734463 |
| ITGB1 | 1410.86 | 766.88 | 1.839740246 |
| PIM1 | 207.59 | 108.8 | 1.907996324 |
| SERPINE1 | 15.38 | 7.96 | 1.932160804 |
| MAPK10 | 5.13 | 2.65 | 1.935849057 |
| GNAS | 8.97 | 4.42 | 2.029411765 |
| BCL3 | 30.75 | 15.04 | 2.044547872 |
| E2F3 | 190.93 | 91.99 | 2.07555169 |
| CTGF | 6.41 | 2.65 | 2.418867925 |
| BCL6 | 11.53 | 4.42 | 2.608597285 |
| TNFSF10 | 105.08 | 39.8 | 2.640201005 |
| BCL2L1 | 15.38 | 5.31 | 2.896421846 |
| MMP1 | 5.13 | 1.77 | 2.898305085 |
| NTRK2 | 5.13 | 1.77 | 2.898305085 |
| EGR1 | 7.69 | 2.65 | 2.901886792 |
| CYP1A1 | 6.41 | 1.77 | 3.621468927 |
| MMP14 | 6.41 | 1.77 | 3.621468927 |
| TIMP1 | 70.48 | 12.38 | 5.693053312 |

**Supplementary Table 2.**

**Differential expression of genes in BCWM.1/IR cells vs. BCWM.1 cells**

**(NanoString nCounter targeted transcriptome analysis)**

| **Gene Name** | **BCWM.1/IR** | **BCWM.1** | **fold change** |
| --- | --- | --- | --- |
| LCK | 2.46 | 86.06 | -34.98373984 |
| SPI1 | 4.92 | 82 | -16.66666667 |
| TGFBI | 0.82 | 11.37 | -13.86585366 |
| FOS | 5.74 | 30.04 | -5.233449477 |
| FGR | 68.92 | 326.37 | -4.735490424 |
| EGR1 | 4.92 | 16.24 | -3.300813008 |
| AREG | 3.28 | 10.55 | -3.216463415 |
| FOSL2 | 4.1 | 12.99 | -3.168292683 |
| PLAUR | 5.74 | 17.05 | -2.970383275 |
| S100A4 | 96.82 | 283.34 | -2.926461475 |
| TNF | 177.23 | 496.05 | -2.798905377 |
| SYK | 54.97 | 153.44 | -2.791340731 |
| ITGB1 | 51.69 | 134.77 | -2.607274134 |
| LIF | 4.1 | 10.55 | -2.573170732 |
| CDKN2A | 4.1 | 9.74 | -2.375609756 |
| SIAH1 | 9.03 | 20.3 | -2.248062016 |
| HCK | 4.1 | 8.93 | -2.17804878 |
| TNFRSF1B | 153.44 | 306.88 | -2 |
| CAV1 | 7.38 | 14.61 | -1.979674797 |
| PTEN | 566.97 | 1085.46 | -1.91449283 |
| EPS8 | 422.56 | 786.69 | -1.861723779 |
| BLM | 103.38 | 176.17 | -1.704101374 |
| TNFRSF10B | 115.69 | 194.03 | -1.677154465 |
| ERBB2 | 4.92 | 8.12 | -1.650406504 |
| JUNB | 114.05 | 177.8 | -1.558965366 |
| BRCA1 | 145.23 | 222.45 | -1.531708325 |
| FAS | 49.23 | 73.88 | -1.500710949 |
| HDAC1 | 1075.69 | 1583.94 | -1.472487427 |
| HSP90AB1 | 8251.89 | 11888.9 | -1.440748725 |
| PDGFA | 28.72 | 40.59 | -1.413300836 |
| PIK3CA | 119.79 | 168.06 | -1.402955172 |
| BRCA2 | 169.03 | 237.06 | -1.402472934 |
| PGK1 | 2874.25 | 4029.27 | -1.401850918 |
| BCL2A1 | 421.74 | 588.6 | -1.395646607 |
| ETS2 | 51.69 | 71.44 | -1.38208551 |
| ERCC2 | 114.87 | 155.88 | -1.357012275 |
| CDK2 | 161.64 | 219.2 | -1.356099975 |
| CCND1 | 42.67 | 57.64 | -1.350831966 |
| FYN | 539.08 | 721.74 | -1.338836536 |
| MYC | 534.15 | 712 | -1.332958907 |
| HPRT1 | 1170.05 | 1557.15 | -1.330840562 |
| NF1 | 166.56 | 220.01 | -1.320905379 |
| BIRC2 | 213.33 | 279.28 | -1.309145455 |
| TGFB1 | 404.51 | 525.27 | -1.298534029 |
| RB1 | 304.41 | 394.56 | -1.296146644 |
| XPC | 173.95 | 224.07 | -1.288128773 |
| BRAF | 117.33 | 151.01 | -1.287053609 |
| IFNGR1 | 234.67 | 302.01 | -1.286956151 |
| CDKN1A | 535.79 | 685.21 | -1.278877919 |
| CCND2 | 1922.46 | 2433.96 | -1.266065354 |
| CHEK1 | 612.1 | 772.89 | -1.262685836 |
| TFE3 | 9.03 | 11.37 | -1.259136213 |
| MTA1 | 677.74 | 839.46 | -1.238616579 |
| BCL3 | 40.21 | 49.52 | -1.231534444 |
| TFDP1 | 909.13 | 1115.5 | -1.226997239 |
| PCNA | 745.03 | 908.47 | -1.219373716 |
| OGG1 | 104.21 | 126.65 | -1.215334421 |
| GAPDH | 15995.07 | 19358.84 | -1.210300424 |
| HRAS | 169.85 | 205.4 | -1.209302326 |
| CD44 | 745.03 | 891.42 | -1.196488732 |
| TNFSF10 | 63.18 | 75.5 | -1.194998417 |
| CCNA2 | 1344 | 1605.86 | -1.19483631 |
| PTPN11 | 388.1 | 463.57 | -1.194460191 |
| TERT | 16.41 | 19.48 | -1.187081048 |
| MLH1 | 310.15 | 366.15 | -1.180557795 |
| GADD45A | 421.74 | 497.67 | -1.180039835 |
| NOTCH1 | 172.31 | 202.97 | -1.177935117 |
| YY1 | 694.97 | 810.24 | -1.165863275 |
| CSK | 856.61 | 994.53 | -1.161006759 |
| AKT2 | 502.97 | 579.67 | -1.152494185 |
| DEK | 1452.31 | 1655.39 | -1.139832405 |
| NPM1 | 32.82 | 37.35 | -1.138025594 |
| LYN | 182.15 | 205.4 | -1.127642053 |
| XRCC5 | 6003.69 | 6753.06 | -1.124818237 |
| TYMS | 4532.51 | 5058.71 | -1.116094614 |
| TFRC | 2249.02 | 2496.47 | -1.1100257 |
| ETV6 | 584.2 | 644.62 | -1.103423485 |
| CDC2 | 1440.82 | 1588.81 | -1.102712344 |
| MSH6 | 292.1 | 321.5 | -1.100650462 |
| HIF1A | 795.08 | 874.37 | -1.099725814 |
| SOD1 | 126.36 | 138.83 | -1.098686293 |
| CASP2 | 329.03 | 361.28 | -1.098015379 |
| TP53 | 550.56 | 596.72 | -1.083841906 |
| DAPK1 | 77.95 | 84.43 | -1.083130212 |
| BCL2 | 172.31 | 185.1 | -1.074226684 |
| YES1 | 103.38 | 110.41 | -1.068001548 |
| IRF1 | 172.31 | 182.67 | -1.060124195 |
| PRKAR1A | 1312 | 1382.6 | -1.053810976 |
| MSH2 | 680.2 | 710.38 | -1.044369303 |
| SFPQ | 2311.38 | 2412.04 | -1.043549741 |
| GUSB | 100.1 | 103.92 | -1.038161838 |
| NRAS | 146.05 | 151.01 | -1.033960972 |
| ERCC4 | 37.74 | 38.97 | -1.032591415 |
| JUN | 20.51 | 21.11 | -1.029254022 |
| NQO1 | 403.69 | 409.99 | -1.015606034 |
| MCL1 | 995.28 | 1009.14 | -1.013925729 |
| DAP3 | 299.49 | 302.01 | -1.008414304 |
| TPR | 351.18 | 353.16 | -1.005638134 |
| MLL | 105.85 | 106.35 | -1.004723666 |
| MPL | 5 | 5 | 1 |
| CCND3 | 85.33 | 85.25 | 1.000938416 |
| BIRC5 | 995.28 | 986.41 | 1.008992204 |
| MPL | 4.1 | 4.06 | 1.009852217 |
| SERPINE1 | 22.97 | 22.73 | 1.010558733 |
| CDK6 | 300.31 | 297.14 | 1.010668372 |
| PTPRG | 9.85 | 9.74 | 1.011293634 |
| STAT3 | 466.05 | 460.33 | 1.012425868 |
| CLTC | 654.77 | 644.62 | 1.015745711 |
| NTRK2 | 126.36 | 124.21 | 1.017309395 |
| TIMP1 | 975.59 | 958 | 1.018361169 |
| RAF1 | 531.69 | 517.97 | 1.061739944 |
| RRM1 | 676.1 | 650.3 | 1.063009127 |
| TUBB | 3268.1 | 3120.8 | 1.066485199 |
| BCR | 414.36 | 394.56 | 1.067342005 |
| CDC25C | 41.85 | 39.78 | 1.075149053 |
| TOP2A | 849.23 | 803.74 | 1.082673703 |
| RAD54L | 218.26 | 206.21 | 1.093826034 |
| REL | 68.1 | 64.14 | 1.116282592 |
| CCNE1 | 216.62 | 203.78 | 1.116655865 |
| E2F1 | 156.72 | 146.95 | 1.118403139 |
| NUMA1 | 185.44 | 173.74 | 1.128529648 |
| FANCG | 164.1 | 152.63 | 1.133152935 |
| IL1B | 12.31 | 11.37 | 1.174434088 |
| HMMR | 215.79 | 197.28 | 1.182898333 |
| CDK4 | 442.26 | 396.19 | 1.200578672 |
| IGF1 | 17.23 | 15.43 | 1.21182266 |
| MYBL2 | 2086.56 | 1865.66 | 1.21182266 |
| TOP1 | 1531.9 | 1357.43 | 1.233490952 |
| E2F3 | 121.44 | 107.17 | 1.256254718 |
| CDKN2C | 35.28 | 30.04 | 1.26249899 |
| PCTK1 | 298.67 | 252.49 | 1.27416332 |
| MYB | 435.69 | 362.9 | 1.299295775 |
| MST1R | 4.92 | 4.06 | 1.34746922 |
| MAPK10 | 21.33 | 15.43 | 1.382372003 |
| PLAT | 4.92 | 4.06 | 1.382372003 |
| APC | 30.36 | 21.92 | 1.385036496 |
| CTNNB1 | 1280.82 | 1038.37 | 1.385036496 |
| TGFBR3 | 36.1 | 25.98 | 1.389530408 |
| ETS1 | 948.51 | 755.03 | 1.389530408 |
| ETV1 | 5.74 | 4.06 | 1.413793103 |
| ABL1 | 312.62 | 247.62 | 1.413793103 |
| STAT1 | 2923.49 | 2009.36 | 1.4549359 |
| RARA | 47.59 | 37.35 | 1.4549359 |
| BMI1 | 355.28 | 239.5 | 1.4834238 |
| MYCN | 7.38 | 5.68 | 1.4834238 |
| BCL2L1 | 22.97 | 15.43 | 1.488658458 |
| WNT10B | 9.85 | 7.31 | 1.488658458 |
| PML | 40.21 | 26.79 | 1.500933184 |
| IL8 | 4.92 | 3.25 | 1.513846154 |
| MAP3K8 | 64 | 40.59 | 1.57674304 |
| FGF2 | 36.1 | 22.73 | 1.588209415 |
| CDC25B | 167.38 | 104.73 | 1.598204908 |
| FGFR3 | 6.56 | 4.06 | 1.615763547 |
| FGFR1 | 45.95 | 27.6 | 1.664855072 |
| PIM1 | 243.69 | 141.26 | 1.725116806 |
| PTK7 | 59.08 | 34.1 | 1.73255132 |
| PTGS2 | 9.85 | 5.68 | 1.73415493 |
| COL1A1 | 52.51 | 30.04 | 1.748002663 |
| KRAS | 392.2 | 223.26 | 1.756696229 |
| ERBB4 | 5.74 | 3.25 | 1.766153846 |
| WNT1 | 4.92 | 2.44 | 2.016393443 |
| FAT1 | 115.69 | 56.83 | 2.03572057 |
| CD34 | 6.56 | 2.44 | 2.68852459 |
| MMP9 | 11.49 | 4.06 | 2.830049261 |
| PDGFRA | 19.69 | 6.49 | 3.033898305 |
| MET | 4.92 | 1.62 | 3.037037037 |
| WT1 | 13.13 | 4.06 | 3.233990148 |
| ABCB1 | 45.13 | 13.8 | 3.270289855 |
| CDH1 | 73.03 | 18.67 | 3.911622924 |
| NGFR | 4.92 | 0.81 | 6.074074074 |
| GNAS | 6.56 | 0.81 | 8.098765432 |
| CDH11 | 46.77 | 4.87 | 9.603696099 |
| THPO | 9.85 | 0.81 | 12.16049383 |
| AR | 18.05 | 0.81 | 22.28395062 |
| CXCL9 | 73.03 | 2.44 | 29.93032787 |
