# Supplementary material for: Waldenstrom macroglobulinemia cells devoid of BTKC481S or CXCR4WHIM-like mutations acquire resistance to ibrutinib through upregulation of Bcl-2 and AKT resulting in vulnerability towards venetoclax or MK2206 treatment
Source: Blood Cancer J. 2017 May 26;7(5):e565–. doi: 10.1038/bcj.2017.40 (PMC5518884; doi:10.1038/bcj.2017.40)
Supplement: Supplementary Materials and Methods [file bcj201740x4.docx]

**Supplementary Materials and Methods**

**Sanger Sequencing of BTK and CXCR4**

RNA was isolated from all WM cells, including ibrutinib-resistant cloens, using the miRCURY RNA isolation kit (300110, Exiqon) and quantified using NanoDrop 3000 (Thermofisher Scientific). Using specific primer sequence for the whole gene; RNA was converted to cDNA by using SuperScript™ III One Step RT-PCR (12574030, Thermofisher Scientific). Primers for BTK and CXCR4 are as below:

BTK Fwd (#BTK-FP-1448-1980)

5’ – TGA ACT ACC TGA GGG AGA TG – 3’

BTK Rev (#BTK-RP-1448-1980)

5’ – TTT CAA GGC CAA AGG AAG TG – 3’

BTK Rev (#BTK-RP-1-507)

5’ – CTC CAA AAT TTG GCA GCC CA – 3’

BTK Fwd (BTK-FP-1-507)

5’ – ATG GCC GCA GTG ATT CTG GA – 3’

BTK Fwd (#BTK-FP-478-987)

5’ – AAA AAT GCT ATG GGC TGC CA – 3’

BTK Rev (#BTK-RP-478-987)

5’ – CCC TTG AGG GTC CCC TGT GG – 3’

BTK Fwd (#BTK-FP-948-1497)

5’ – AGT GTC TGT GTT TGC TAA AT – 3’

BTK Rev (#BTK-RP-948-1497)

5’ – TAG CAG CTG CTG AGT CTG GA – 3’

CXCR4 Fwd Set 1

5’ – CAA GAC CAC AGT CAT CCT CAT C – 3’

CXCR4 Rev Set 1

5’ – CCA CGA GAC ATA CAG CAA CTA A – 3’

CXCR4 Fwd Set 2

5’ – GAA GCC CAG AAT GGT TTG TAT TT – 3’

CXCR4 Rev Set 2

5’ – GCT GTA GAG GTT GAC TGT GTA G– 3’

CXCR4 Fwd Set 3

5’ – GAG AAG CAT GAC GGA CAA GTA – 3’

CXCR4 Rev Set 3

5’ – TGA CAA TAC CAG GCA GGA TAA G – 3’

CXCR4 Fwd Set 4

5’ – CAA ACG CGC CAA GTG ATA AA– 3’

CXCR4 Rev Set 4

5’ – GGG TTC CTT CAT GGA GTC ATA G– 3’

CXCR4 Rev Set 5

5’ – AGT CCT ACC ACG AGA CAT ACA – 3’

**Whole Exome Sequencing**

DNA samples from BCWM.1, MWCL-1 and RPCI-WM1 and their respective ibrutinib-resistant derivatives (BCWM.1/IR, MWCL-1/IR, RPCI-WM1/IR), were captured using SureSelect Human All Exon V5+UTRs from Agilent. Additional details are described in the supplementary materials and methods section. Captured samples were sequenced on the Illumina HiSeq2000, and 122-227 million, 101bp paired-end reads were generated. FASTQC was applied to check base quality, GC content, and read length distribution. Data were then analyzed by Mayo Clinic’s in-house GenomeGPS v2.0 bioinformatics pipeline. 120-223 million reads were mapped to GRCh37 human reference genome using Novoalign v2.08. 92-176 million reads were mapped to the 74.86Mbp capture region, giving more than 20X coverage for more than 90% of the capture region. GATK v2.7 was used to call variants. Integrative Genomics Viewer (IGV) was used to visualize reads aligned to given genomic regions.

**Calculation of Drug Combination Effects**

Drug combination synergy, additivity or antagonism were determined using the CompuSyn software (ComboSyn Inc., Paramus, NJ, USA), which employs the Chou-Talalay principle (1). We treated WM cells with different concentrations of ibrutinib, venetoclax or MK2206 for 48hr in quadruplicate followed by measurement of viability using the CellTiter Glo assay, according to the manufacturers instructions. Cell viability data were expressed as the fraction of anti-proliferative activity (Fraction affected, Fa) by the individual drugs or the combination in drug-treated cells from which a combination index (CI) value was derived. An additive effect is indicated by a CI of 1, an antagonistic effect is denoted by CI >1 and a synergistic effect is indicated by a CI <1.

**References.**

1. Chou TC, Talalay P. Quantitative analysis of dose-effect relationships: the combined effects of multiple drugs or enzyme inhibitors. Advances in enzyme regulation. 1984;22:27-55.
